# CovEMERALD: Assessing the feasibility and preliminary effectiveness of remotely delivered Eye Movement Desensitisation and Reprocessing following Covid-19 related critical illness: A structured summary of a study protocol for a randomised controlled trial

**DOI:** 10.1186/s13063-020-04805-1

**Published:** 2020-11-17

**Authors:** Andrew Bates, Sophie Rushbrook, Elan Shapiro, Michael Grocott, Rebecca Cusack

**Affiliations:** 1 University Hospital Southampton NHS Foundation Trust, Southampton, UK; 2 University of Southampton, Southampton, UK; 3 Dorset Healthcare University NHS Foundation Trust, Poole, UK; 4 Independent Consultant, Haifa, Israel

**Keywords:** COVID-19, Randomised controlled trial, Protocol, Eye-movement desensitisation and reprocessing, EMDR, Intensive Care, Survivors, Psychology, PTSD, Anxiety

## Abstract

**Objectives:**

**Primary Objective:** To determine the feasibility of delivering a protocolised, remote, online, Eye Movement Desensitisation and Reprocessing (EMDR) intervention, within 12-weeks of hospital discharge, for adult survivors of Covid-19 related critical illness.

**Secondary objectives:** To investigate whether remotely delivered EMDR can improve psychological outcome following Covid-19 related critical illness, specifically Post-Traumatic Stress Disorder (PTSD), anxiety and depression.

**Trial design:**

This is a single centre, randomised controlled cohort feasibility trial.

**Participants:**

Participants will be recruited following discharge from the Intensive Care Unit at University Hospital Southampton, United Kingdom. Eligible patients will have received mechanical ventilation for a minimum of 24 hours, tested Covid-19 positive by polymerase chain reaction, will be over the age of 18 years and have the capacity to provide informed consent. Patients will be excluded if they have pre-existing cognitive impairment, pre-existing psychotic diagnosis or are not expected to survive post-hospital discharge.

**Intervention and comparator:**

Group one: patients in the control arm will receive their standard package of prescribed care, following discharge home from hospital. If they experience any adverse physical or psychological health-conditions, they will access care through the usual available channels.

Group two: patients randomly allocated to the intervention arm will receive their standard package of prescribed care, following discharge home from hospital. In addition, they will be referred to the Intensive Psychological Therapies Service in Poole, United Kingdom. They will receive an online appointment within 12-weeks of discharge home from hospital. They will receive a maximum of eight, weekly sessions of EMDR, delivered by a trained psychological therapist, following the Recent Traumatic Episode Protocol (R-TEP). Appendices 1 and 2 of the attached trial protocol contain a detailed description of the R-TEP intervention, written in accordance with the Template for Intervention Description and Replication (TIDieR) checklist and guide.

**Main outcomes:**

The primary outcome from this trial will be feasibility. Feasibility will be determined by recruitment rates, expressed as a percentage of eligible patients approached, completion of the EMDR intervention, completion of final assessment at 6-months, incidence of attributable adverse events and protocol adherence by the psychological therapists.

Secondary, exploratory outcomes will be assessed by comparison between the control and intervention groups at 6-months post-hospital discharge. Psychometric evaluation will consist of the PTSD Checklist-Civilian Version and Hospital Anxiety and Depression Scale. In addition, we will assess health-related quality of life using the EQ5D-5L, physical activity using wrist worn activity monitors and nutritional state using the Council of Nutrition Appetite Questionnaire.

**Randomisation:**

Consenting participants will be randomly allocated to intervention or usual care using an internet-based system (ALEA^TM^). Participants will be randomly assigned, on a 1:1 ratio, to receive either standard care (control) or the standard care plus online EMDR R-TEP (Intervention)

**Blinding (masking):**

Due to the nature of the intervention, participants cannot be blinded to group allocation. 6-month patient reported outcome measures will be completed using an online, electronic case report form. Group allocation will be masked during data analysis.

**Numbers to be randomised (sample size):**

This is a feasibility study, the results of which will be used to power a definitive study if appropriate. We anticipate a 25% mortality /loss to follow-up. A total of 26 patients will be recruited to this study, 13 patients in each arm.

**Trial Status:**

CovEMERALD opened to recruitment on 23^rd^ September 2020 with an anticipated recruitment period of 6-months. We are using protocol version number 1.2 (1^st^ June 2020)

**Trial registration:**

CovEMERALD was registered on clinicaltrials.gov NCT04455360 on 2^nd^ July 2020

**Full protocol:**

The full protocol is attached as an additional file, accessible from the Trials website (Additional file 1). In the interest in expediting dissemination of this material, the familiar formatting has been eliminated; this letter serves as a summary of the key elements of the full protocol.

The study protocol has been reported in accordance with the Standard Protocol Items: Recommendations for Clinical Interventional Trials (SPIRIT) guidelines (Additional file 2).

## Supplementary information

**Additional file 1.** Full study protocol.

**Additional file 2.** SPIRIT 2013 Checklist: Recommended items to address in a clinical trial protocol and related documents

## Data Availability

All staff will act to preserve patient confidentiality and will not disclose any information by which patients may be identified. Electronic copies of the CRF will be transferred using secure nhs.net email accounts, with data encrypted to ensure anonymity. All procedures for handling, storing, destroying and processing data will be compliant with the Data Protection Act 2018. All trial documentation and data will be archived centrally by the Sponsor at the end of trial in a purpose designed facility for ten years in accordance with regulatory requirements. Access to these archives will be restricted to authorised personnel. Electronic data sets will be stored indefinitely.

