# Supplementary material for: CovEMERALD: Assessing the feasibility and preliminary effectiveness of remotely delivered Eye Movement Desensitisation and Reprocessing following Covid-19 related critical illness: A structured summary of a study protocol for a randomised controlled trial
Source: Trials. 2020 Nov 17;21:929. doi: 10.1186/s13063-020-04805-1 (PMC7670988; doi:10.1186/s13063-020-04805-1)
Supplement: Supplementary file 1 — Additional file 1. Full study protocol. [file 13063_2020_4805_MOESM1_ESM.docx]

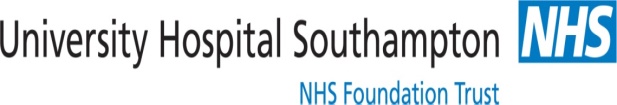

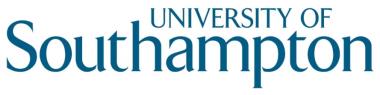


**CovEMERALD:**

**Can an online, Eye Movement Desensitisation and Reprocessing intervention improve psychological outcome following Covid-19 related critical illness: A feasibility trial**

| Protocol version: | v1.2 |
| --- | --- |
| Protocol date: | 1^st^ June 2020 |
| Co- Chief investigator and trial co-ordinator:  Co-investigator:  Key investigators:  Public/ patient investigators: | Andrew Bates  Critical Care Nurse Research Manager  NIHR BRC Anaesthesia and Critical Care Research  University Hospital Southampton NHS foundation Trust  Tremona Road  Southampton  SO16 6YD  Tel: 023 8120 6317  e-mail: a.bates@soton.ac.uk  Dr Rebecca Cusack  Consultant, General Intensive Care Unit  University Hospital Southampton NHS foundation Trust  Tremona Road  Southampton  SO16 6YD  Tel: 023 8120 5308  e-mail: [Rebecca.cusack@uhs.nhs.uk](mailto:Rebecca.cusack@uhs.nhs.uk)  Dr Sophie Rushbrook, Consultant Clinical Psychologist, Head of Intensive Psychological Therapies Service, Dorset Healthcare University NHS Foundation Trust, Branksome, Poole  Elan Shapiro, Psychologist, EMDR Europe Accredited Consultant  Prof Mike Grocott, Professor of Anaesthesia and Critical Care University Hospital Southampton  Roddy Wyatt and Jason Durno |
| Sponsor details: | University Hospital Southampton  RHM CRI0397 |
| NHS REC Committee number: | 20/HRA/3633 |
| IRAS Project ID: | 256821 |
| ClinicalTrials.gov Register Number | NCT04455360 |

Trial Management

For general enquiries please contact:

Andrew Bates

Research Manager,

National Institute for Health Research, Biomedical Research Council, Critical Care,

University Hospital Southampton NHS Foundation Trust,

Tremona Road,

Southampton.

SO16 6YD

a.bates@soton.ac.uk

Tel: 023 8120 6317

Table of Contents

[1. Trial synopsis 4](#_Toc44686768)

[1. List of abbreviations 5](#_Toc44686769)

[2. Study team 6](#_Toc44686770)

[3. Study sites 6](#_Toc44686771)

[6](#_Toc44686772)

[4. Abstract 7](#_Toc44686773)

[5. Lay summary 8](#_Toc44686774)

[6. Background and rationale 9](#_Toc44686775)

[7. Aims 13](#_Toc44686776)

[Primary aim: 13](#_Toc44686777)

[Secondary objectives: To investigate whether online EMDR R-TEP affects 13](#_Toc44686778)

[Figure 1: Trial patient flow 14](#_Toc44686779)

[8. Method 15](#_Toc44686780)

[*8.1* *Trial Design and setting* 15](#_Toc44686781)

[*8.2* *Patient eligibility* 15](#_Toc44686782)

[*8.3* *Recruitment and consent process* 15](#_Toc44686783)

[8.4 *Patient randomisation* 15](#_Toc44686784)

[*8.5* *Intervention* 15](#_Toc44686785)

[*8.6* *Assessments* 16](#_Toc44686786)

[**Primary Outcome measure** 16](#_Toc44686787)

[**Secondary outcome measures** 16](#_Toc44686788)

[*8.7* *Additional data* 17](#_Toc44686789)

[9. Sample size 19](#_Toc44686790)

[10. Process evaluation 19](#_Toc44686791)

[11. Safety considerations 19](#_Toc44686792)

[12. Data collection and confidentiality 20](#_Toc44686793)

[13. Withdrawal of patient consent 21](#_Toc44686794)

[14. Monitoring and trial oversight 21](#_Toc44686795)

[15. Ethics 22](#_Toc44686796)

[16. Sponsorship 22](#_Toc44686797)

[17. Dissemination plan 22](#_Toc44686798)

[References 23](#_Toc44686799)

[Appendix One: 28](#_Toc44686800)

[The Recent Traumatic Episode Protocol (R-TEP): An Integrative Protocol for Early EMDR Intervention (EEI) 28](#_Toc44686801)

[Appendix 2: 31](#_Toc44686802)

[CovEMERALD Online EMDR R-TEP for survivors of Covid-19 relayed critical illness: Template for intervention description and replication (TIDieR) checklist and guide [75]. 31](#_Toc44686803)

1. **Trial synopsis**

| **Title** | Can an online, Eye Movement Desensitisation and Reprocessing intervention improve psychological outcome following Covid-19 related critical illness: A Covid-19 targeted feasibility trial |
| --- | --- |
| **Acronym** | CovEMERALD |
| **Inclusion criteria** | - Acute admission to Intensive Care receiving invasive ventilation for at least 24 hours - Covid-19 positive by PCR - >18 years of age on date of ICU admission - Capacity to provide informed consent and to complete trial interventions and assessments |
| **Exclusion criteria** | - Pre-existing cognitive impairment such as dementia - Pre-existing psychotic diagnosis - Refusal to grant consent |
| **Sponsor reference number** | RHM CRI0397 |
| **Trial design** | Single-centre feasibility study of a two-arm randomised controlled trial |
| **Participation number** | 26 patients |
| **Rationale** | A number of contemporaneous studies have reported Covid-19 related mental health issues. By 19^th^ June 2020 over 12,000 patients, with confirmed Covid-19, had been admitted to UK Intensive Care Units.  Patients who survive a period of critical illness have a disproportionately high chance of suffering from significant and persistant poor psychological outcomes.  Our study programme aims to investigate whether it is feasible to deliver an online Eye Movement Desensitisation Reprocessing (EMDR) intervention, delivered soon after hospital discharge and whether this intervention will improve psychological outcome for survivors of Covid-19 related critical illness. |
| **Study aims** | To evaluate the feasibility of delivering an online early EMDR protocol to patients who have survived Covid-19 related critical illness. |
| **Intervention** | Trial particpants, randomised to the intervention arm, will undertake an online psychological therapy EMDR Recent Tramatic Events Protocol (R-TEP).  This will consist of 2-8 personal sessions of EMDR R-TEP delivered by a suitably trained and experienced psychotherapist. |
| **Control arm** | Trial particpants randomised to the control arm will receive usual post-hospital discharge care. |
| **Feasibility outcomes** | 1. Able to recruit >30% of eligible patients approached 2. Complete early EMDR intervention programme in 75% or more of trial participants randomised to intervention. 3. Protocol adherence 4. Incidence of attributable adverse events 5. Outcome measures completed in 75% or more of trial participants |

1. **List of abbreviations**

| ASD | Acute Stress Disorder |
| --- | --- |
| BLS | Bilateral stimulation |
| BPS | British Psychological Society |
| BRS | Brief Resilience Scale |
| CBT | Cognitive Behavioural Therapy |
| CNAQ | Council on Nutrition Appetite Questionnaire |
| Covid-19 | Coronavirus Disease 2019 |
| EMDR | Eye Movement Desensitisation and Reprocessing |
| EQ5D-5L | EuroQol Five Dimension- Five level scale |
| HADS | Hospital Anxiety and Depression Scale |
| ICNARC | Intensive Care National Audit and Research Centre |
| ICU | Intensive Care Unit |
| IPTS | Intensive Psychology Therapy Services |
| MERS | Middle Eastern Respiratory Syndrome |
| MOCA | Montreal Cognitive Assessment |
| NHSx | National Health Service digital health and social care programme - |
| PCL-C | PTSD Checklist – Civilian Version |
| PCR | Polymerase Chain Reaction |
| PGH | Poole General Hospital |
| PHEIC | Public Health Emergency of International Concern |
| PTSD | Post-Traumatic Stress Disorder |
| RBCH | Royal Bournemouth and Christchurch Hospital |
| RCT | Randomised Controlled Trial |
| R-TEP | Recent traumatic Event Protocol |
| SARS-CoV-2 | Severe Acute Respiratory Syndrome Coronavirus |
| SUD | Subjective Units of Disturbance |
| UHS | University Hospital Southampton |
| UK | United Kingdom |
| WHO | World Health Organisation |

1. **Study team**

| **Andrew Bates**  Research Manager Critical Care  NIHR Biomedical Research Council  Southampton University Hospital NHS FT /University of Southampton NIHR / BRC Critical Care Research Area  **Dr Rebecca Cusack**  Honorary Senior Lecturer in Critical Care Medicine  Southampton University Hospital NHS FT /University of Southampton NIHR / BRC Critical Care Research Area  **Dr Sophie Rushbrook**  Consultant Clinical Psychologist, Head of Intensive Psychological Therapies Service, Dorset Healthcare University NHS Foundation Trust, Branksome, Poole  **Prof Michael Grocott**  Professor of Anaesthesia, Peri-operative and Critical Care Medicine  Southampton University Hospital NHS FT /University of Southampton NIHR / BRC Critical Care Research Area  **Elan Shapiro**  Psychologist, EMDR Europe Accredited Consultant | |
| --- | --- |
| Patient representatives | Mr Roddy Wyatt  Mr Jason Durno |
| Trial management | Mr Andrew Bates Research Manager Critical Care  NIHR Biomedical Research Council  Southampton University Hospital NHS FT /University of Southampton NIHR / BRC Critical Care Research Area  a.bates@soton.ac.uk |

1. **Study sites**

**Recruitment sites:**

University Hospital Southampton, Tremona Road, Southampton. SO16 6YD

**Intervention site:**

Intensive Psychological Therapies Service, Branksome Clinic, 51A Layton Road, Poole, BH12 2BJ

1. **Abstract**

**Background:** Since the emergence of Covid-19, over 12,000 patients have been admitted to UK Intensive Care Units with a confirmed diagnosis. Primarily a disease of the respiratory tract, many of these patients are experiencing mechanical ventilation and ICU length of stay, more than 4 times as long as non-Covid-19 ICU patients. Data from previous coronavirus epidemics report anxiety, depression and PTSD in up to 44% of survivors, persisting beyond 12-months post disease recovery. These psychological pathologies can be persistent and have been linked with increased morbidity and mortality. Due to the emergent nature of Covid-19, no long-term psychological outcome data exist, however post-ICU psychopathology is associated with length of mechanical ventilation, delirium and length of ICU stay. A 2020 expert consensus group predicted that up to 30% of Covid-19 survivors will require psychological intervention.

Eye-Movement Desensitisation and Reprocessing (EMDR) has reduced the incidence of psychological morbidity in war veterans and victims of man-made and natural disasters. Small studies have also shown it to be effective in healthcare settings, within the Emergency department, following cancer diagnosis and implantation of cardioverter defibrillators. EMDR is validated by NICE guidance for use in treating adult onset PTSD. The EMDR Recent-Traumatic Events protocol (R-TEP) enables an individual to process memories of the event in order to reduce psychological morbidity. EMDR R-TEP should be delivered within 3-months of the onset of a traumatic event. Social distancing guidelines and the potentially long-term nature of the Covid-19 epidemic require the adoption and robust testing of technological solutions, in order to assure access to best possible psychological care. Such an approach is supported by NHSx, the British Psychological Society and the American Psychiatric Association.

**Aims:** To ensure compliance with social-distancing guidelines, CovEMERALD will investigate whether it is feasible to deliver 2-8 online sessions of EMDR R-TEP, starting within 12-weeks of hospital discharge, for adult survivors of Covid-19 related critical illness. In addition, we will investigate whether the online EMDR R-TEP intervention reduces patient reported psychological symptoms.

These data will inform the design of a future randomised control trial , investigating the efficacy of an early EMDR R-TEP intervention in reducing psychological morbidity for Covid-19 related critical care survivors.

**Method:** Hospital-based research staff will approach consecutive eligible patients in hospital or within 12-weeks of hospital discharge to discuss the trial and to present the Patient Information Sheet. Consenting patients will complete baseline assessments, before random allocation to treatment or usual care. Patients in the intervention arm will be referred to an NHS psychological therapy service, whose staff are trained and experienced EMDR R-TEP practitioners. The psychological therapy service will arrange for an online, virtual baseline visit. There will be a minimum of 2 and a maximum of 8 sessions depending on patient need, assessed by the treating psychological practitioner, as per the EMDR R-TEP protocol. Each session lasts for 60-90 minutes. When the practitioner assesses that the patient is no longer experiencing disturbing thoughts or memories, the intervention will be complete.

The patient will be asked to repeat the assessments, 6-months following discharge from hospital. In addition, we will complete a post-study, process evaluation, consisting of semi-structured interviews with a representative sample of participants and staff.

**Outcomes:** Primary outcome will be feasibility and tolerability of the intervention. This will be assessed by recruitment rates and adherence to the EMDR R-TEP intervention and follow-up assessments.

Exploratory data of effectiveness will be collected by patient-reported questionnaire. We will assess PTSD symptoms using the PTSD CheckList – Civilian Version (PCL-C). We will assess anxiety and depression using the Hospital Anxiety and Depression Scale (HADS) and EuroQol Five Dimension- Five level scale (EQ5D-5L). The Montreal Cognitive Assessment (MOCA) tests cognitive ability. Patients will be asked to wear an activity monitor for 7-days. The Council of Nutrition Appetite Questionnaire (CNAQ) and Brief Resilience Scale (BRS) will also be completed at baseline and 6-months post-discharge.

The process evaluation will reveal themes that we will use to assess fidelity, quality and implementation.

These data will inform the design of a larger multi-centre follow-up study investigating the clinical effectiveness of an early EMDR intervention, for adult survivors of Covid-19 related critical illness.

1. **Lay summary**

The Covid-19 epidemic has resulted in a significant number of patients being admitted to Intensive Care for life-saving treatment. Unfortunately, experience from previous global epidemics and from Intensive Care survivors, shows us that a life-threatening illness and subsequent life-saving treatment, in the Intensive Care Unit can lead to disabling and long-lasting psychological problems.

A large, recent study showed that around 50% of Intensive Care survivors will suffer from anxiety, depression and/or post-traumatic stress disorder. Studies which followed up previous survivors of epidemics show similar rates of psychological distress. These conditions can persist for an extended period of time, significantly reduce the patient’s quality of life and are linked with long-term physical health problems. These problems occur more frequently in Intensive Care survivors than in the average population and more frequently than people suffering from cancer or serious heart disease.

When patients have been discharged from Intensive Care, in the UK, there is very little follow-up care or specialist rehabilitation. Only 27% of Intensive Care units contact their patients after discharge while only 6% offer any sort of physical or psychological therapy. Even for hospitals that do offer follow-up care, there is little agreement on what psychological therapies may be effective for this group of patients.

A therapy called Eye-Movement Desensitisation and Reprocessing (EMDR) is recommended, by NHS expert guidelines, for treating Post-Traumatic Stress Disorder and can be effective for anxiety and depression, linked to traumatic, life-threatening events. It has been particularly well-studied in soldiers returning from war and victims of earthquakes, fires and assaults. More recently, some studies have shown that it can also help people to come to terms with life-threatening health conditions such as cancer and heart disease. One very small study has suggested that it may be effective for Intensive care survivors.

To ensure adherence with social distancing guidelines, our expert psychological therapy team have begun to deliver the EMDR therapy via online videoconferencing. This has been shown to be equally as effective as face-to-face sessions and is supported by the NHS technology arm, British Psychological Society and the American Psychiatric Association. The additional advantage is that ICU survivors will be spared a journey to a treatment centre and can undertake the therapy at home or the location of their choosing. Because we think that this is worthy of extra investigation, we aim to determine whether it is feasible to deliver online EMDR therapy to survivors of Covid-19 related critical illness and whether it might help to reduce psychological trauma associated with the illness and treatment.

A total of 26 survivors of intensive care, will be offered the EMDR therapy, in order to see whether they feel it is a good idea and whether they will volunteer to undertake sessions with a highly trained and experienced team of psychological therapists. These patients will be randomly placed into one of two groups.

In the ‘intervention group’, 13 patients will be referred to a local NHS community-based EMDR therapy team, run by expert Clinical Psychologists. They will attend between 2 and 8 online sessions, starting within 12-weeks of hospital discharge. We will ask our patients to complete some survey questionnaires, before and after therapy, to see whether it makes them feel better about their stressful experience. We will also record whether the patients completed the therapy and how they felt it might be improved or made more accessible.

In the ‘control group’, the remaining 13 patients will undertake all of the same questionnaires, but will not receive online EMDR. Patient’s final involvement will be to repeat the questionnaires 6-months after they have been discharged from hospital.

When the trial has been completed, we will ask to interview a number of trial participants and staff involved in the trial. The information that we gain from this study will be used to design a much larger study, which will investigate whether EMDR can be effective for all survivors of intensive care.

1. **Background and rationale**

In December 2019, the Wuhan Municipal Health Committee in China reported an emergent outbreak of viral pneumonia of unknown aetiology. Chinese scientists quickly identified and shared the genetic sequence of a new type of Severe Acute Respiratory Syndrome Coronavirus, SARS-CoV-2. The associated disease was named Covid-19. On 30^th^ January 2020 the World Health Organisation declared a Public Health Emergency of International Concern (PHEIC). The disease burden has been particularly high within Europe. By the end of May 2020, the United Kingdom had reported 39,045 deaths, the second highest total in the world, behind the United States.

During this period, 12,086 patients with biologically confirmed Covid-19, have been admitted to UK Intensive Care Units. Of the 7786 patients whose data have been submitted to the Intensive Care National Audit and Research Centre (ICNARC), 5761 (74%) have received advanced respiratory support via mechanical ventilation. Median ICU length of stay for Covid-19 survivors has been 11 days, compared to 6 days for survivors of non-Covid-19 viral pneumonia and 2.4 days for all admissions in 2018-2019. [1]

Intensive care units in the National Health Service (NHS) in the UK, admitted over 290,000 patients in 2018/9[2]. As advances in medical treatment have improved survival from critical illness, we are becoming increasingly aware that many survivors experience substantial physical, cognitive and psychological impairment[3].[4-6] These effects can persist for years and often result in a reduced quality of life with significantly social and economic burdens for both the patient and their relatives. [7-9]

**Psychological symptoms following critical illness:**

It has been estimated that approximately 50% of critically ill patients suffer serious emotional distress, and up to two-thirds have unusual experiences such as hallucinations and delusions, while in intensive care. [10, 11] Emotional distress, including severe symptoms of anxiety, low mood and panic, may be caused by a range of stressful experiences that are common in the intensive care: fear of dying; invasive treatments such as mechanical ventilation; pain and discomfort; inability to communicate; and terrifying hallucinatory delusions.[10-12][13].The hallucinations and delusions of intensive care unit patients have been linked to delirium, the provision and withdrawal of sedative and other psychoactive drugs, effects of illness (such as sepsis), immobility, and sensory and sleep deprivation [11, 14, 15].

These experiences are among identified risk factors for longer-term post-critical care post-traumatic stress disorder (PTSD), depression, anxiety or cognitive impairment[11] [16-18].

**Psychological symptoms following coronavirus epidemics:**

In the last 20 years there have been two global epidemics of coronavirus related respiratory diseases, SARS-CoV-1 and the Middle Eastern Respiratory Syndrome (MERS), with high levels of anxiety, depression and PTSD among survivors [19].

Anxiety, depression and/ or PTSD remained present at 12-months post-recovery for up to 44% of SARS patients [20] and 42% of MERS patients.[21]

Due to the emerging stage of the disease, no data exist for longer-term psychological outcomes of Covid-19 survivors. However, data from previous global epidemics, the disease severity combined with extended length of ICU stay make it reasonable to hypothesise that this group are vulnerable to suffering significant and persistent psychopathologies. Based on existing data, the 2020 Stanford Hall consensus statement suggested that up to 30% of Covid-19 survivors will experience incomplete processing of their experience, requiring psychological intervention. [22]

**Post-Traumatic Stress Disorder (PTSD)**

The diagnosis of acute stress disorder (ASD) can be considered 3 days to a month after experiencing a traumatic event. ASD requires meeting the criteria for at least 9 of 14 symptoms[23]. Acute stress while in the ICU and early memories of frightening ICU experiences (e.g paranoid delusions, hallucinations and nightmares) have been identified as independent risk factors for long term psychological morbidity [10]. It is postulated that acute stress disorder may result from fragmented memories of traumatic experiences, [11] and can develop into PTSD when symptoms persist for more than a month[23, 24]

Post-traumatic stress disorder (PTSD) is defined, in accordance with the DSM-5, as present if an individual is exposed to actual or threatened serious injury/death (e.g., critical illness and related ICU treatments) and subsequently develops the following symptoms which last more than 1 month and cause significant distress or changes in functionality: persistently re-experiencing the event and attempting to avoid trauma-related stimuli; new negative alterations in mood/cognition; and new/increased arousal/reactivity[23].

The incidence of PTSD reported in the adult general population of the UK is 3% [25] and double this in war veterans [26]. Medical event induced PTSD has become recognised over the past 20 years Higher rates are also reported in patients diagnosed with serious cardiovascular disease (12%) [27] and cancer (13%). [28].

Estimates of the prevalence of PTSD in ICU survivors have a wide range. This may be attributable to variability in patient cohort, instruments used to diagnose PTSD and timing of assessment[12, 18, 29, 30]. Systematic reviews of survivors of intensive care identified high rates of PTSD (median 20%)[12] [31] and depression (median 30%),[32, 33] lasting for months or years after leaving intensive care. In the UK, every year this equates to 58,000 patients suffering PTSD symptoms and 87,000 patients with depression following an episode of critical illness. Patients who develop serious long-term psychological distress are at much higher risk of associated physical morbidities and mortality [34, 35].

**Individual and public health cost:**

For survivors of Intensive Care, PTSD, anxiety and depression are associated with physical morbidity including musculoskeletal pain, hypertension, obesity, and cardiovascular disease [36, 37] . Patients with symptoms of depression are 47% more likely to die during the first two years after intensive care discharge.[38]. Following an ICU admission patients have increased use of healthcare resources leading to 50% higher NHS costs per patient in the five years following intensive care [9, 39]

**Why does PTSD occur?**

It is believed that memories are stored in networks of related information. Activating one aspect of this network may trigger associated memories and emotions. Over time these memories should be processed and become neutral. If this occurs in a normal manner a traumatic event can be recalled without associated feelings of arousal, fear and anxiety. However, when recalling the event is too upsetting, an individual may be unable to process and store the memory in a coherent narrative that reflects the removal of the original threat. This can result in an ongoing and repetitive stress response.[40, 41]. Risk factors identified for developing post ICU PTSD include mechanical ventilation, use of benzodiazepines for sedation, pre-existing PTSD and depression, delirium within the ICU[12] [42] [43]

**Treating PTSD**

There are a large number of pharmacological and non-pharmacological treatments for PTSD. UK guidance for treating PTSD from the National Institute for Health and Care Excellence [44]recommends the use of either Cognitive Behavioural Therapy (CBT) or Eye Movement Desensitisation and Reprocessing (EMDR). There is however very limited investigation into the prevention of PTSD after traumatic experiences.

**Early interventions**

With the current understanding that ASD can proceed PTSD there has been a move towards delivering interventions early – before the symptoms fulfil the prerequisite of being present of a month before PTSD can be diagnosed. Evidence now supports that brief psychological interventions (5 sessions) may be effective if treatment starts within the first month after the traumatic event. Beyond the first month, the duration of treatment is similar to that for chronic PTSD.[45]

Attempts to prevent the development of PTSD psychological debriefing (PD) have previously been assessed, [46] however studies have demonstrated worse outcomes in patients who have PD compared to controls [47, 48] and a Cochrane review states ‘compulsory debriefing of trauma victims should cease’ [49].

Early cognitive behavioural therapy (CBT) is reported to be of benefit in the treatment of ASD [50]. A recent randomised controlled study demonstrated that both CBT and prolonged exposure interventions reduced PTSD symptoms at 5 months if commenced at 4 weeks post trauma in patients with symptoms ASD [51].

CBT is a talking therapy which aims to help patients manage their problems by addressing thought and behaviours. Multi-facetted intervention including CBT in acutely ill trauma survivors requiring surgery, improved PTSD symptoms and physical function was seen at one year [52]. In this study, interventions were commenced within days or weeks of the trauma event. CBT has also been shown to prevent PTSD in acute trauma patients by 50%. More recent studies when CBT is delivered within weeks or months support these findings [50] [53]

In 2019, the POPPI study reported that a CBT-based technique, delivered in the ICU by trained nurses, was not effective in reducing incidence of PTSD, anxiety or depression following intensive care.[54]

In a study by Rothbaum, early intervention, with prolonged exposure therapy, following a traumatic experience reduced the progression of stress symptoms into PTSD. These patients presenting to a hospital emergency department commenced treatment in the majority of cases (88%) within 24 hours of the presenting trauma [55]

While the burden of PTSD in critical illness survivors has gained increasing recognition in recent years, relatively little work has been completed, examining treatments for PTSD due to acute life-threatening illnesses

EMDR is a form of psychotherapy treatment whereby the client verbally relates a narrative of a traumatic episode or emotionally disturbing material in brief sequential doses while simultaneously focusing on an external stimulus. Therapist directed repeated lateral eye movements are the most commonly used external stimulus but a variety of other stimuli can be used. The aim is to help patients process the fragmented traumatic memories associated with PTSD. This can help patients to remember the trauma without experiencing such upsetting symptoms.[56] (Shapiro, 1997) Despite increasing demonstration of its effectiveness there is limited knowledge of the underlying mechanism of action, [57] however neurobiological effects of EMDR have been reported by brain imaging before and after treatment[58].

A number of studies and meta-analyses have reported EMDR’s effectiveness in treating PTSD, depression and anxiety in adults who have experienced life-threatening trauma [59-62]. and has repeatedly demonstrated better outcomes in reducing PTSD and anxiety symptoms compared to other forms of treatment such as Cognitive Behaviour Therapy (CBT), relaxation therapy, and Prolonged Exposure (PE)[63-65].

EMDR has reduced psychological symptoms in patients who have had unavoidable traumatic experiences during medical care such associated with implantable cardioverter defibrillators (ICD) shocks following cardiac issues and intensive cancer treatments.[64, 66]. Research informing the best timing to provide psychological interventions for trauma associated with medical care suggests that post hospital discharge (8-weeks )[15] or at outpatient follow-up clinics [67] may be too late, and earlier intervention could be more beneficial. [68]. A number of systematic reviews have reported no adverse events attributable to EMDR (52-56).

**EMDR Recent-Traumatic Events Protocol:**

In patients who have experienced critical illness the traumatic episode can be regarded as the continuum of all that has happened from the initial traumatic event, until the present day covering the entirety of the ICU admission, up to the point of therapy.

Systematic reviews suggest that earlier interventions appeared to be more effective at preventing long-term psychological symptoms before they become entrenched[24, 69]. The POPPI study which examined delivering psychological support to the patient whilst still being care for within the ICU did not show benefit. It is suggested that the intervention may have been too early with the patients being too ill and fatigued to implement some of the suggested behavioural strategies [54].

The EMDR Recent Traumatic Events Protocol (R-TEP) is designed to be delivered soon after the traumatic event, in order to promote memory processing that will be preventative against longer-term psychological symptoms[70]. EMDR R-TEP conceptualises the memories as unprocessed experiences which are still fragmented due to the recent and potentially ongoing nature of the trauma.

In 2018, Gil-Jardine et al reported that an EMDR R-TEP intervention, delivered in the Emergency Room, for patients presenting with a medical or traumatic event, reduced incidence of PTSD at 3-months. PTSD symptoms were experienced by 3% of patients who had received EMDR R-TEP compared with 19% of the patients who did not receive EMDR R-TEP [71]. EMDR R-TEP has not been used in patients at risk of PTSD following critical illness.

**Online psychological care:**

Social distancing guidelines and the potentially long-term nature of the Covid-19 epidemic require the adoption and robust testing of technological solutions, in order to assure access to best possible psychological care.

The American Psychiatric Association Donald M. Hilty, 2020 #568} and the British Psychological Society[72] support online videoconferencing to offer psychological support, based on considerations of safety, increased access and effectiveness [73] .

Our psychology team are already delivering EMDR R-TEP online. This approach is supported by the NHS digital arm NHSx.[74]

1. **Aims**

## Primary aim:

This is a feasibility study to test whether an early online EMDR R-TEP intervention, can be delivered, starting within 12-weeks following hospital discharge, for adult survivors of Covid-19 related critical illness.

## Secondary objectives: To investigate whether online EMDR R-TEP affects

1. Psychological symptoms
2. Quality of life
3. Cognitive function
4. Physical activity
5. Nutritional status

A subsequent process evaluation will be conducted to assess therapeutic adherence to the EMDR protocol and to gather opinion and evidence of participants. Our results, will inform the design of a future randomised-controlled trial to test the effectiveness of the online EMDR R-TEP intervention in ameliorating psychological problems for adult survivors of Covid-19 related critical illness.

# **Figure 1: Trial patient flow**


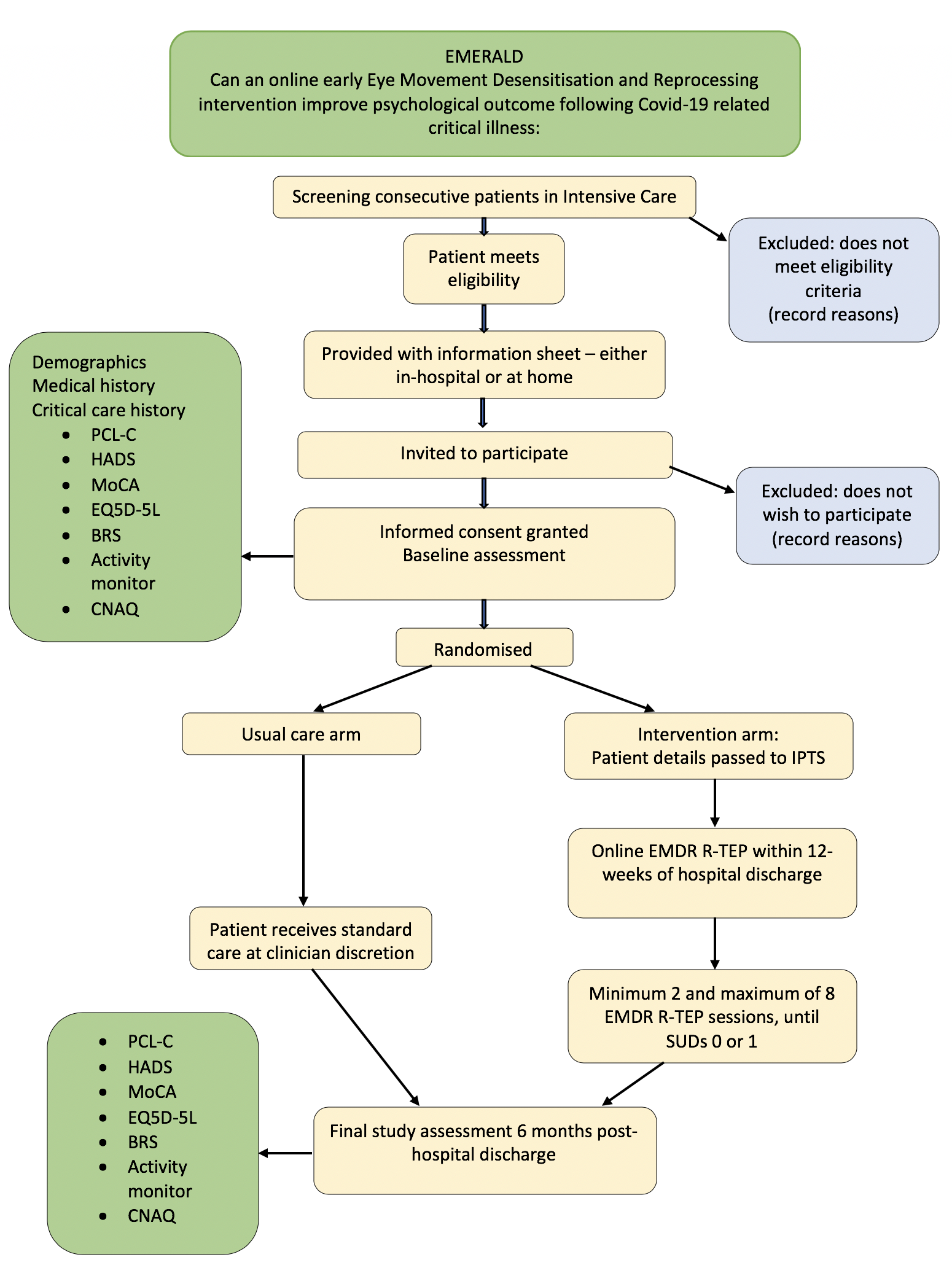


1. **Method**
   1. ***Trial Design and setting***

This is a single-centre, randomised controlled feasibility cohort trial.

Patients will be recruited from University Hospital Southampton Intensive Care Unit on the south coast of the United Kingdom.

Consenting participants will be referred to IPTS, Branksome Clinic, a community-based, NHS specialist therapy support unit, for people with emotional and psychological health problems.

- 1. ***Patient eligibility***

**Inclusion Criteria:**

Acute admission to Critical Care who have required mechanical ventilation for at least 24 hours

Covid-19 positive by PCR

>18 years of age

Capacity to provide informed consent and to complete trial interventions and assessments.

**Exclusion criteria:**

Pre-existing cognitive impairment such as dementia

Pre-existing psychotic diagnosis

Not expected to survive post-hospital discharge

- 1. ***Recruitment and consent process***

Hospital research staff will screen all patients within the Intensive Care Unit (ICU). Patients who fulfil the inclusion criteria and have none of the exclusion criteria will be approached to discuss trial participation either within the hospital or within 12-weeks of hospital discharge. Capacity to consent must be determined in accordance to the Mental Capacity Act 2005.

In-hospital approach: The initial conversation can be made face-to-face within the hospital. The patient will be given the Patient Information Sheet and adequate time to consider participation according to Good Clinical Practice guidelines.

Prior to hospital discharge, hospital research staff, with delegated duty of consent, will ask the patient to consider signing an informed consent form provided by the trial.

Post-hospital discharge approach: If the patient has been discharged from hospital, within the last 12-weeks, research staff will make initial contact with a telephone call. If the patient expresses an interest, research staff will post or email the study Patient Information Sheet. A follow-up phone call will be arranged. If the patient expresses a desire to participate in the study, the research staff will document the conversation and sign a consent form on behalf of the patient. A copy of the consent form will be posted to the patient.

- 1. ***Patient randomisation***

Consenting participants will be randomly allocated to intervention or usual care using an internet based system. Participants will be randomly assigned to receive either standard care (control) or the standard care plus online EMDR R-TEP (Intervention) [70]

- 1. ***Intervention***

Group one: patients in the control arm will receive their standard package of prescribed care, following discharge home from hospital. If they experience any adverse physical or psychological health-conditions they will access care through the usual available channels.

Group two: patients in the intervention arm will receive their standard package of prescribed care, following discharge home from hospital. In addition they will be referred to the Intensive Psychology Therapy Service. They will receive an initial online appointment within 12-weeks of discharge home from hospital. Their assigned psychological therapist will introduce the therapy and assess their distress using the 'Subjective Units of Distress' tool. Patients rate their distress on a scale from 0 (no anxiety at all) to 10 (unbearably bad).

The therapist will then ask the patient to recount the story of their Intensive Care stay, while watching the therapist's finger-movements or tapping, in a side-to-side motion. The science behind this is not fully understood, but it is believed to help patients to mentally process traumatic events, while remaining calm. Traumatic events negatively affect people, in the long-term, when memories of them have not been fully or effectively processed.

Following the online EMDR R-TEP sessions, the therapist will repeat the subjective units of distress assessment. If, after 2-sessions, the patient has subjective units of distress, related to the Intensive Care stay, at 0/10 (Peace, serenity, total relief) or 1/10 (No acute distress and feeling basically good), then the intervention will be complete and no more sessions are needed. This is consistent with the EMDR R-TEP protocol, developed by our trial group member. If the subjective units of distress are higher than 1 at the end of a session, the patient will be asked to come back again, repeating sessions, to a maximum of 8 or until they score 0/10 or 1/10.

If after 8-sessions of EMDR R-TEP, the patient is experiencing ongoing distress the therapy team will arrange an appropriate package of NHS care.

If at any time, other psychological issues arise or the patient exhibits signs of considerable distress, the therapy team, including the lead Consultant Clinical Psychologist, will arrange appropriate psychological care.

Please refer to appendices 1 and 2 for full details describing the EMDR R-TEP intervention, according to the Template for Intervention description and replication (TIDieR) checklist.[75.]

- 1. ***Assessments***

**Primary Outcome measure**

**Feasibility**

Hospital research staff will collect data on number of eligible patients from their ICU, number of eligible patients approached and number consented.

Therapy staff will collect data on adherence to the trial protocol and sessions attended.

Feasibility will be determined by:

- Recruitment of >30% of eligible patients approached
- Complete early EMDR intervention programme in 75% or more of trial participants
- Protocol adherence
- Incidence of attributable adverse events
- Outcome measures completed in 75% or more of trial participants

**Secondary outcome measures**

All secondary outcome measures will be collected at baseline and 6-months post-hospital discharge

**Post-traumatic stress disorder:**

The PTSD Checklist-Civilian Version (PCL-C) is a validated, standardised self-reporting questionairre for PTSD comprising of 17 items that correspond to key PTSD symptoms[77]. Using a 5-point scale, participants are asked to indicate how much they have been bothered by these problems within the last month. (1= not at all; 5 = Extremely) PCL-C is rated by adding total scores.

**Anxiety and depression:** Hospital Anxiety and Depression Scale (HADS) is a 14-item, self-reported measure with 7-items relating to symptoms of anxiety and 7-items relating to depression[78]. Each item is scored from 0-3, providing categorical total scores for anxiety and depression. 0-7 = normal. 8-10 = borderline. 11-21 = abnormal.

**Cognitive function:** Montreal Cognitive Assessment (MoCA) is a validated tool, used to detect cogntive impairment[79]. MoCA is a 30-point test of short-term memory, visuospatial ability, executive function, attention, language, abstract reasoning and orientation. Scores range from 0-30. A score higher than 26 is considered to indicate normal cognitive function.

**Health Related Quality of Life:** EQ5D -5L comprises five quality-of-life dimensions; mobility, self-care, usual activities, pain/discomfort andanxiety/depression. Participants report levels ranging from ‘no problems’ to ‘extreme problems’.

**Resilience:** The Brief Resilience Scale is a validated measure of resilience. The capacity to recover from a traumatic event is dependent on mental processing of those trauma.

**Physical activity:** Patients will wear a wristband activity monitor following consent and at final assessment point, 6-months post-hospital discharge. The equipment has been validated for medical use and will be worn 24 hours per day for 7 consecutive days.

**Nutritional assessment:** The scored Council of Nutrition Appetite Questionnaire provides a validated assessment of appetite and predicted weight-loss and takes approximately 5-minutes to complete.

- 1. ***Additional data***

Hospital research staff will provide the following demographic and hospital admission information:

Age

Gender

Living arrangements

Postcode

Ethnicity

Employment status

Pre admission functional comorbidity index

ICU diagnosis

APACHE II score [81]

Duration of level 3 care

Duration of level 2 care

ICU and hospital length of stay

Days of ventilation

Sedation days

Episodes of delirium according to daily CAM-ICU measures[82]

|  | **Baseline** | **Weekly – starting within 12-weeks of hospital discharge** | **6-months post-hospital discharge** | **Post-study completion** |
| --- | --- | --- | --- | --- |
| **Demographics** | **X** |  |  |  |
| **Medical history** | **X** |  |  |  |
| **Admission data** | **X** |  |  |  |
| **Informed consent** | **X** |  |  |  |
| **PCL-5** | **X** |  | **X** |  |
| **HADS** | **X** |  | **X** |  |
| **Montreal Cognitive Assessment** | **X** |  | **X** |  |
| **EQ5D-5L** | **X** |  | **X** |  |
| **BRS** | **X** |  | **X** |  |
| **CNAQ** | **X** |  | **X** |  |
| **Activity monitor** | **X** |  | **X** |  |
| **EMDR R-TEP Intervention** |  | **X** |  |  |
| **SUDs** |  | **X** |  |  |
| **Post-session assessment** |  | **X** |  |  |
| **Semi-structured interview^*^** |  |  |  | **X** |

**Figure two: trial schedule of events**

^*^ Representative sample of particpants and staff

1. **Sample size**

This is a feasibility study, the results of which will be used to power a definitive study if appropriate. We anticipate a 25% mortality /loss to follow-up. A total of 26 patients will be recruited to this study, with estimated total of 18-20 patients completing the study.

1. **Process evaluation**

Qualitative, in-depth, semi-structured interviews will be conducted with patients enrolled in the trial and professionals involved in the delivery of the trial at completion. Patients from both arms of the study (up to 6) and professionals from participating sites (up to 6) will be interviewed. This will provide an opportunity for the research team to reflect on the experiences of patients and professionals, identifying barriers and facilitators to trial processes (professionals) and trial experiences (patients), in order to gain an in-depth understanding on process/relationships/communications that helped or hindered conduct of the trial.

Within the self-report questionnaire patients will be asked to indicate whether they can be contacted to participate in an interview. Interviews will be conducted by telephone. Interviews will be conducted by a researcher with experience in qualitative process evaluations. Encrypted audio recorders will be used.

1. **Safety considerations**

A number of systematic reviews have reported no adverse events attributable to EMDR (52-56). The intervention will be undertaken by suitably trained and experienced psychological practitioners from the NHS Intensive Psychology Therapy Service, Branksome Clinic in Poole. IPTS primarily provides a service to individuals with complex and severe, persistent psychopathologies. The service has an established and defined risk management and clinical governance structure.

All interventions will be delivered or supervised by Dr Sophie Rushbrook, Consultant Clinical Psychologist, Head of Service and trial board member.

Online sessions will be compliant with Digital Approaches to therapy guidance from the British Psychological Society [72]and NHS Digital [74]. (These guidance contain expected standards relating to safeguarding, information governance, and GDPR.

Prior to starting the EMDR R-TEP therapy the psychological therapist will undertake a risk assessment process. They will discuss who is present with the participant, whether support is available and ensure that confidentiality is maintained. In addition, emergency numbers (GP and 999) will be made available.

Participants who exhibit symptoms of intrusion/ escalation will be treated according to the protocol unless it is determined that further treatment or escalation to emergency care may be necessary/ indicated. If further treatment is required, the most appropriate course of action and referral pathway will be decided on a case-by-case basis by the psychology team.

Only adverse events (see definitions below) which occur during the intervention phase of the trial will be documented. These will be graded according to CTCAE version 4.0. Medical judgement will be exercised to determine whether an AE is serious. AEs that are not serious according to the definitions below but may jeopardise the subject or may require intervention to prevent a SAE, should also be considered as serious and reported accordingly.

All serious adverse events (SAE) will be documented and reported to the trial sponsor at [sponsor@uhs.nhs.uk](mailto:sponsor@uhs.nhs.uk) within 24 hours of occurrence.

Any deviations from the planned protocol will be recorded and reported immediately to the trial sponsor at [sponsor@uhs.nhs.uk](mailto:sponsor@uhs.nhs.uk).

Complete the SAE/SUSAR form & email a scanned copy of the form with as many details as possible to the sponsor (sponsor@uhs.nhs.uk) together with anonymised relevant treatment forms and investigation reports.
Or
Contact the Research Ethics Committee by phone for advice and then email a scanned copy of the completed SAE/SUSAR form.

The UHS team will notify the necessary REC of all SUSARs occurring during the trial within 15 days. All investigators will be informed of all SUSARs occurring throughout the trial.

Local investigators should report any SUSARs and /or SAEs as required by their Local Research Ethics Committee and/or Research & Development Office.

**Adverse Event (AE):** any untoward medical occurrence in a participant or clinical trial subject that does not necessarily have a causal relationship with trial treatment or participation.

**Serious Adverse Event (SAE):** any untoward medical occurrence or effect that at any dose:

- Results in death
- Is life-threatening – refers to an event in which the subject was at risk of death at the time of the event; it does not refer to an event which hypothetically might have caused death if it were more severe
- Requires hospitalisation, or prolongation of existing in participants’ hospitalisation
- Results in persistent or significant disability or incapacity

**Suspected Unexpected Serious Adverse Reaction (SUSAR):** any suspected adverse reaction related to the trial procedures that is both unexpected and serious.

1. **Data collection and confidentiality**

Demographic and baseline questionnaire data will be collected following consent. Research nurses at the recruiting site will contact the participants via telephone at 4-months post-hospital discharge (+/- 2-weeks). Participants will be invited to repeat the baseline questionnaires, which will be posted to them with a pre-paid envelope. If participants prefer, the questionnaires can be completed over the telephone. These data will be securely stored as paper case report forms at the recruiting site.

All entries must be clear, legible and concordant with Good Clinical Practice. The local Principle Investigator is responsible for accuracy of data. Participating staff must be listed on the signed delegation log.

Prior to commencing the online conversation the therapy team will reiterate the boundaries of confidentiality and ensure ongoing consent. The participant’s preference of Skype^TM^ or Zoom^TM^ will be used in accordance with NHS Digital guidance. The therapist will not record the videoconference, unless specifically asked to do so by the participant, in which case written consent will need to be attained and the recording will be treated as part of their medical record. As in any other consultation, the therapist will ensure GDPR compliance for data storage and processing according to NHS Digital standards. [74]

The CovEMERALD study team and participating sites will act concordant with the Medical Research Council’s Guidelines for Good Clinical Practice in Clinical Trials and Good Research Practice: Principles and Guidelines.

All staff will act to preserve patient confidentiality and will not disclose any information by which patients may be identified. Electronic copies of the CRF will be transferred using secure nhs.net email accounts, with data encrypted to ensure anonymity. All procedures for handling, storing, destroying and processing data will be compliant with the Data Protection Act 2018.

All trial documentation and data will be archived centrally by the Sponsor at the end of trial in a purpose designed facility for ten years in accordance with regulatory requirements. Access to these archives will be restricted to authorised personnel. Electronic data sets will be stored indefinitely.

1. **Withdrawal of patient consent**

Patients who consent to trial participation, consent to undertake the assessments, interventions, follow-up and data collection. Trial participants retain the right to withdraw consent to trial participation at any point. This decision will be respected and the trial sponsor will be notified in writing. Participants will be asked whether the study team may continue to use trial data previously collected. If this is denied, all trial data will be destroyed in accordance with GDPR and Good Clinical Practice. Details will be recorded in the patient’s medical notes and no further data collection will occur.

1. **Monitoring and trial oversight**

This protocol, patient information sheets, informed consent forms and additional trial related literature will be reviewed by a Research Ethics Committee for compliance with applicable clinical research regulations. Approval will be gained before study commencement.

Day to day management of CovEMERALD, including site set-up, training and urgent consideration of safety concerns, will be the joint responsibility of Andrew Bates, Sophie Rushbrook and Dr Rebecca Cusack, (Co-Investigators) with additional supervisory oversight from Professor Mike Grocott.

**Trial Management Group:** bi-monthly meetings to discuss trial progress, issues arising and related research. Members include Andrew Bates, Rebecca Cusack, Sophie Rushbrook, Elan Shapiro, Mike Grocott and patient representatives Roddy Wyatt and Jason Durno.

**Trial sponsorship:** CovEMERALD will be peer-reviewed by subject expert clinicians and sponsored by University Hospital Southampton NHS Foundation Trust. Protocol deviations / violations and serious breaches of GCP will be reported to the sponsor using [sponsor@uhs.nhs.uk](mailto:sponsor@uhs.nhs.uk)

**Monitoring:** The CI and PIs will facilitate any local monitoring by the R&D quality manager, REC review and provide access to source data as required. Following any monitoring a report will be provided which will summarise the visit and documents, along with any findings. The CI will be responsible for ensuring that all findings are addressed appropriately. The study group will review all events in a timely manner. Additional monitoring will be scheduled where there is evidence of suspicion of non-compliance with the Study protocol.

1. **Ethics**

After HRA approval, and the site has confirmed capacity & capability to deliver the study. Each site will be activated before patients can be entered into the trial.

The trial will be conducted in accordance with the recommendations for physicians involved in research on human subjects adopted by the 18th World Medical Assembly, Helsinki 1964 as revised and recognised by governing laws and EU Directives. Each subject’s consent to participate in the trial should be obtained after a full explanation has been given of treatment options, including the conventional and generally accepted methods of treatment. The right of the subject to refuse to participate in the trial without giving reasons must be respected.

After the subject has entered the trial, the clinician may give alternative treatment to that specified in the protocol, at any stage, if they feel it to be in the best interest of the subject. However, reasons for doing so should be recorded and the subject will remain within the trial for the purpose of follow-up and data analysis according to the treatment option to which they have been allocated. Similarly, the subject remains free to withdraw at any time from protocol treatment and trial follow-up without giving reasons and without prejudicing their further treatment.

1. **Sponsorship**

University Hospital Southampton NHS Foundation Trust is acting as the sponsor for this trial. UHS Research and Development will ensure that all regulatory policies adhered to in line with GCP. Other delegated duties will be assigned to the NHS Trusts or others taking part in this trial by means of the site clinical trial agreement. Any trial protocol deviations/violations and breaches of Good Clinical Practice occurring at sites should be reported to [sponsor@uhs.nhs.uk](mailto:sponsor@uhs.nhs.uk) and the local R&D Office immediately. The sponsor will then advise of and/or undertake any corrective and preventative actions as required.

The trial may be subject to inspection and audit by University Hospital Southampton NHS Foundation Trust, as the Sponsor’s delegate and other regulatory bodies to ensure adherence to ICH GCP, UK Policy for Health and Social Care Research, applicable contracts/agreements and national regulations.

The sponsor of the project is University Hospital Southampton NHS Foundation Trust. For NHS sponsored research HSG (96) 48 reference no.2 refers. If there is negligent harm during the clinical project when the NHS body owes a duty of care to the person harmed, NHS Indemnity covers NHS staff, medical academic staff with honorary contracts, and those conducting the project. NHS Indemnity does not offer no-fault compensation and is unable to agree in advance to pay compensation for non-negligent harm. Ex-gratia payments may be considered in the case of a claim.

1. **Dissemination plan**

Results of this proposed feasibility study will be disseminated with the aim of demonstrating feasibility of the intervention. In addition we hope to stimulate enthusiasm for centres to participate in the intended future trial.

Four key audiences for dissemination have been identified: i) patients and public; ii) Intensive care staff, healthcare workers and potential future research delivery partners; iii) service delivery organisations and iv) academic and potential future research collaborators.

Dissemination activities will include but not be limited to:

- Publication in peer reviewed journal
- Feedback to PPI study focus group
- Feedback to study participants
- Presentations to local clinical teams and managers and commissioners
- Presentation at conferences attended by appropriate healthcare professionals

Authors will be the chief investigator, principal investigators and members of the research team, each individually named.

If appropriate workshops will be organised to bring together stakeholders to share and interpret the findings with the aim of contributing to future trial design.

**References**

1. ICNARC, *ICNARC report on COVID-19 in critical care 29 May 2020*. 2020, Intensive Care National Audit and Research centre: London.

2. *Hospital Admitted Patient Care Activity 2018-19*. 2019; National stastics]. Available from: <https://digital.nhs.uk/data-and-information/publications/statistical/hospital-admitted-patient-care-activity/2018-19>.

3. Esteban, A., et al., *Evolution of Mortality over Time in Patients Receiving Mechanical Ventilation.* American Journal of Respiratory and Critical Care Medicine, 2013. **188**(2): p. 220-230.

4. Kaukonen, K.M., et al., *Mortality Related to Severe Sepsis and Septic Shock Among Critically III Patients in Australia and New Zealand, 2000-2012.* Jama-Journal of the American Medical Association, 2014. **311**(13): p. 1308-1316.

5. Iwashyna, T.J., et al., *Long-term Cognitive Impairment and Functional Disability Among Survivors of Severe Sepsis.* Jama-Journal of the American Medical Association, 2010. **304**(16): p. 1787-1794.

6. Herridge, M.S., et al., *Functional Disability 5 Years after Acute Respiratory Distress Syndrome.* New England Journal of Medicine, 2011. **364**(14): p. 1293-1304.

7. Cuthbertson, B.H., et al., *Quality of life in the five years after intensive care: a cohort study.* Critical Care, 2010. **14**(1).

8. Lone, N.I., et al., *Five-Year Mortality and Hospital Costs Associated with Surviving Intensive Care.* American Journal of Respiratory and Critical Care Medicine, 2016. **194**(2): p. 198-208.

9. Griffiths, J., et al., *An exploration of social and economic outcome and associated health-related quality of life after critical illness in general intensive care unit survivors: a 12-month follow-up study.* Critical Care, 2013. **17**(3).

10. Wade, D.M., et al., *Investigating risk factors for psychological morbidity three months after intensive care: a prospective cohort study.* Critical Care, 2012. **16**(5).

11. Jones, C., et al., *Precipitants of post-traumatic stress disorder following intensive care: a hypothesis generating study of diversity in care.* Intensive Care Medicine, 2007. **33**(6): p. 978-985.

12. Wade, D., et al., *Identifying clinical and acute psychological risk factors for PTSD after critical care: a systematic review.* Minerva Anestesiologica, 2013. **79**(8): p. 944-963.

13. Novaes, M., et al., *Stressors in ICU: perception of the patient, relatives and health care team.* Intensive Care Medicine, 1999. **25**(12): p. 1421-1426.

14. Ely, E.W., et al., *The impact of delirium in the intensive care unit on hospital length of stay.* Intensive Care Medicine, 2001. **27**(12): p. 1892-1900.

15. Jones, C., et al., *Memory, delusions, and the development of acute posttraumatic stress disorder-related symptoms after intensive care.* Critical Care Medicine, 2001. **29**(3): p. 573-580.

16. Granja, C., et al., *Posttraumatic stress disorder-related symptoms after critical care: The role of sedation and family Reply.* Critical Care Medicine, 2009. **37**(5): p. 1832-1833.

17. Myhren, H., et al., *Patients' memory and psychological distress after ICU stay compared with expectations of the relatives.* Intensive Care Medicine, 2009. **35**(12): p. 2078-2086.

18. Davydow, D.S., et al., *A longitudinal investigation of posttraumatic stress and depressive symptoms over the course of the year following medical-surgical intensive care unit admission.* General Hospital Psychiatry, 2013. **35**(3): p. 226-232.

19. Gardner, P.J. and P. Moallef, *Psychological Impact on SARS Survivors: Critical Review of the English Language Literature.* Canadian Psychology-Psychologie Canadienne, 2015. **56**(1): p. 123-135.

20. Tansey, C.M., et al., *One-Year Outcomes and Health Care Utilization in Survivors of Severe Acute Respiratory Syndrome.* Archives of Internal Medicine, 2007. **167**(12): p. 1312-1320.

21. Lee, S.H., et al., *Depression as a Mediator of Chronic Fatigue and Post-Traumatic Stress Symptoms in Middle East Respiratory Syndrome Survivors.* Psychiatry investigation, 2019. **16**(1): p. 59-64.

22. Barker-Davies, R.M., et al., *The Stanford Hall consensus statement for post-COVID-19 rehabilitation.* British Journal of Sports Medicine, 2020: p. bjsports-2020-102596.

23. Association, A.P., *DMS-5*. 2013: Americian Psychiatric Association.

24. Roberts, M.B., et al., *Early Interventions for the Prevention of Posttraumatic Stress Symptoms in Survivors of Critical Illness: A Qualitative Systematic Review.* Critical Care Medicine, 2018. **46**(8): p. 1328-1333.

25. Greenberg, N., S. Brooks, and R. Dunn, *Latest developments in post-traumatic stress disorder: diagnosis and treatment.* British Medical Bulletin, 2015. **114**(1): p. 147-155.

26. Stevelink, S.A.M., et al., *Mental health outcomes at the end of the British involvement in the Iraq and Afghanistan conflicts: a cohort study.* British Journal of Psychiatry, 2018. **213**(6): p. 690-697.

27. Vilchinsky, N., et al., *Cardiac-disease-induced PTSD (CDI-PTSD): A systematic review.* Clin Psychol Rev, 2017. **55**: p. 92-106.

28. Hahn, E.E., et al., *Post-traumatic stress symptoms in cancer survivors: relationship to the impact of cancer scale and other associated risk factors.* Psycho-oncology, 2015. **24**(6): p. 643-652.

29. Jackson, J.C., et al., *Post-traumatic stress disorder and post-traumatic stress symptoms following critical illness in medical intensive care unit patients: assessing the magnitude of the problem.* Critical Care, 2007. **11**(1).

30. Griffiths, J., et al., *The prevalence of post traumatic stress disorder in survivors of ICU treatment: a systematic review.* 2007. **33**(9): p. 1506-1518.

31. Righy, C., et al., *Prevalence of post-traumatic stress disorder symptoms in adult critical care survivors: a systematic review and meta-analysis.* Critical Care, 2019. **23**.

32. Davydow, D.S., et al., *Symptoms of Depression in Survivors of Severe Sepsis: A Prospective Cohort Study of Older Americans.* American Journal of Geriatric Psychiatry, 2013. **21**(9): p. 887-897.

33. Bienvenu, O.J., et al., *Depressive Symptoms and Impaired Physical Function after Acute Lung Injury A 2-Year Longitudinal Study.* American Journal of Respiratory and Critical Care Medicine, 2012. **185**(5): p. 517-524.

34. Ballenger, J.C., *Recognizing the patient with social anxiety disorder.* International Clinical Psychopharmacology, 2000. **15**: p. S1-S5.

35. Boscarino, J.A., *Psychobiologic predictors of disease mortality after psychological trauma - Implications for research and clinical surveillance.* Journal of Nervous and Mental Disease, 2008. **196**(2): p. 100-107.

36. Eddleston, J.M., P. White, and E. Guthrie, *Survival, morbidity, and quality of life after discharge from intensive care.* Critical Care Medicine, 2000. **28**(7): p. 2293-2299.

37. McFarlane, A.C., *The long-term costs of traumatic stress: intertwined physical and psychological consequences.* World Psychiatry, 2010. **9**(1): p. 3-10.

38. Hatch, R., et al., *Anxiety, Depression and Post Traumatic Stress Disorder after critical illness: a UK-wide prospective cohort study.* Critical Care, 2018. **22**.

39. Lone, N.I., et al., *Surviving Intensive Care: A Systematic Review of Healthcare Resource Use After Hospital Discharge.* Critical Care Medicine, 2013. **41**(8): p. 1832-1843.

40. van der Kolk, B.A., *The psychology and psychobiology of developmental trauma.* Praxis Der Kinderpsychologie Und Kinderpsychiatrie, 1998. **47**(1): p. 19-35.

41. van Der Kolk, B.A. and R. Fisler, *Dissociation and the fragmentary nature of traumatic memories: Overview and exploratory study.* Journal of Traumatic Stress, 1995. **8**(4): p. 505-525.

42. Girard, T.D., et al., *Risk factors for post-traumatic stress disorder symptoms following critical illness requiring mechanical ventilation: a prospective cohort study.* Critical Care, 2007. **11**(1).

43. Patel, M.B., et al., *Incidence and Risk Factors for Intensive Care Unit-related Post-traumatic Stress Disorder in Veterans and Civilians.* American Journal of Respiratory and Critical Care Medicine, 2016. **193**(12): p. 1373-1381.

44. Excellence, N.I.o.H.a.C., *Post Traumatic stress disorder (NG116)*. 2018.

45. Bryant, R.A., et al., *Treating acute stress disorder: An evaluation of cognitive behavior therapy and supportive counseling techniques.* American Journal of Psychiatry, 1999. **156**(11): p. 1780-1786.

46. Litz, B.T., et al., *Early intervention for trauma: Current status and future directions.* Clinical Psychology-Science and Practice, 2002. **9**(2): p. 112-134.

47. McNally, R.J., Bryant, R. ,Ehlers,A., *DOES EARLY PSYCHOLOGICAL*

*INTERVENTION PROMOTE RECOVERY*

*FROM POSTTRAUMATIC STRESS?* American Psychological Society, 2003. **4**: p. 45-79.

48. Sijbrandij, M., et al., *Emotional or educational debriefing after psychological trauma. Randomised controlled trial.* The British journal of psychiatry : the journal of mental science, 2006. **189**: p. 150-155.

49. Rose, S.C., et al., *Psychological debriefing for preventing post traumatic stress disorder (PTSD).* Cochrane Database of Systematic Reviews, 2002(2).

50. Bryant, R.A., et al., *Treatment of acute stress disorder: A comparison of cognitive-behavioral therapy and supportive counseling.* Journal of Consulting and Clinical Psychology, 1998. **66**(5): p. 862-866.

51. Shalev, A.Y., et al., *Prevention of Posttraumatic Stress Disorder by Early Treatment.* Archives of General Psychiatry, 2012. **69**(2): p. 166-176.

52. Zatzick, D., et al., *A Randomized Stepped Care Intervention Trial Targeting Posttraumatic Stress Disorder for Surgically Hospitalized Injury Survivors.* Annals of Surgery, 2013. **257**(3): p. 390-399.

53. Bryant, R.A., et al., *The Additive Benefit of Hypnosis and Cognitive-Behavioral Therapy in Treating Acute Stress Disorder.* Journal of Consulting and Clinical Psychology, 2005. **73**(2): p. 334-340.

54. Wade, D.M., et al., *Effect of a Nurse-Led Preventive Psychological Intervention on Symptoms of Posttraumatic Stress Disorder Among Critically Ill Patients A Randomized Clinical Trial.* Jama-Journal of the American Medical Association, 2019. **321**(7): p. 665-675.

55. Rothbaum, B.O., et al., *Early Intervention May Prevent the Development of Posttraumatic Stress Disorder: A Randomized Pilot Civilian Study with Modified Prolonged Exposure.* Biological Psychiatry, 2012. **72**(11): p. 957-963.

56. Shapiro, F., *Eye movement desensitization: A new treatment for post-traumatic stress disorder.* Journal of Behavior Therapy and Experimental Psychiatry, 1989. **20**(3): p. 211-217.

57. Landin-Romero, R., et al., *How Does Eye Movement Desensitization and Reprocessing Therapy Work? A Systematic Review on Suggested Mechanisms of Action.* Frontiers in Psychology, 2018. **9**.

58. Boukezzi, S., et al., *Grey matter density changes of structures involved in Posttraumatic Stress Disorder (PTSD) after recovery following Eye Movement Desensitization and Reprocessing (EMDR) therapy.* Psychiatry Research-Neuroimaging, 2017. **266**: p. 146-152.

59. Chen, Y.R., et al., *Efficacy of Eye-Movement Desensitization and Reprocessing for Patients with Posttraumatic-Stress Disorder: A Meta-Analysis of Randomized Controlled Trials.* Plos One, 2014. **9**(8).

60. Calancie, O.G., et al., *Eye movement desensitization and reprocessing as a treatment for PTSD: current neurobiological theories and a new hypothesis.* Annals of the New York Academy of Sciences, 2018. **1426**(1): p. 127-145.

61. Wilson, G., et al., *The Use of Eye-Movement Desensitization Reprocessing (EMDR) Therapy in Treating Post-traumatic Stress Disorder-A Systematic Narrative Review.* Frontiers in Psychology, 2018. **9**.

62. Moreno-Alcazar, A., et al., *Efficacy of Eye Movement Desensitization and Reprocessing in Children and Adolescent with Post-traumatic Stress Disorder: A Meta-Analysis of Randomized Controlled Trials.* Frontiers in Psychology, 2017. **8**.

63. Arabia, E., M.L. Manca, and R.M. Solomon, *EMDR for survivors of life-threatening cardiac events: Results of a pilot study.* Journal of EMDR Practice and Research, 2011. **5**(1): p. 2-13.

64. Capezzani, L., et al., *EMDR and CBT for cancer patients: Comparative study of effects on PTSD, anxiety, and depression.* Journal of EMDR Practice and Research, 2013. **7**(3): p. 134-143.

65. Ironson, G., et al., *Comparison of two treatments for traumatic stress: A community-based study of EMDR and prolonged exposure.* Journal of Clinical Psychology, 2002. **58**(1): p. 113-128.

66. Peregrinova, L. and B. Hamann, *Eye movement desensitization and reprocessing in psychocardiological settings. Trauma-specific processing of shock due to implantable cardioverter-defibrillators.* Psychotherapeut, 2017. **62**(6): p. 520-527.

67. Cuthbertson, B.H., et al., *The PRaCTICaL study of nurse led, intensive care follow-up programmes for improving long term outcomes from critical illness: a pragmatic randomised controlled trial.* BMJ, 2009. **339**(oct16 1): p. b3723-b3723.

68. Peris, A., et al., *Early intra-intensive care unit psychological intervention promotes recovery from post traumatic stress disorders, anxiety and depression symptoms in critically ill patients.* Critical Care, 2011. **15**(1): p. R41.

69. Wade, D. and V. Page, *Long-Term Mental Health After ICU, Let's Go Through the Looking Glass.* Critical Care Medicine, 2016. **44**(10): p. 1934-1935.

70. Shapiro, E. and B. Laub, *The recent-traumatic episode protocol (R-TEP): An integrative protocol for early EMDR intervention (EEI)*, in *Eye Movement Desensitization and Reprocessing (EMDR) Scripted Protocols: Basics and Special Situations*, M. Luber, Editor. 2009, New York, NY: Springer Publishing Co. p. 251-68.

71. Gil-Jardine, C., et al., *Prevention of post-concussion-like symptoms in patients presenting at the emergency room, early single eye movement desensitization, and reprocessing intervention versus usual care: study protocol for a two-center randomized controlled trial.* Trials, 2018. **19**.

72. Society, B.P., *Adaptations to psychological practice: Interim guidance during Covid-19 pandemic*. 2020: Leicester, UK.

73. Bashshur, R.L., et al., *The Empirical Evidence for Telemedicine Interventions in Mental Disorders.* Telemedicine and E-Health, 2016. **22**(2): p. 87-113.

74. NHSx, *COVID-19 Information governance advice for IG professionals*. 2020.

75. Hoffmann, T.C., et al., *Better reporting of interventions: template for intervention description and replication (TIDieR) checklist and guide.* BMJ : British Medical Journal, 2014. **348**: p. g1687.

76. Weiss, D.S., & Marmar, C. R, *The Impact of Event Scale - Revised*, in *Assessing psychological trauma and PTSD*, T.M.K. J. Wilson Editor. 1996, New York: Guilford. p. 399-411.

77. Blevins, C.A., et al., *The Posttraumatic Stress Disorder Checklist for DSM-5 (PCL-5): Development and Initial Psychometric Evaluation.* Journal of Traumatic Stress, 2015. **28**(6): p. 489-498.

78. Zigmond, A.S. and R.P. Snaith, *The Hospital Anxiety and Depression Scale.* Acta Psychiatrica Scandinavica, 1983. **67**(6): p. 361-370.

79. Nasreddine, Z.S., et al., *The Montreal Cognitive Assessment, MoCA: A Brief Screening Tool For Mild Cognitive Impairment.* Journal of the American Geriatrics Society, 2005. **53**(4): p. 695-699.

80. Üstün, T.B. and W.H. Organisation, *Measuring health and disability: Manual for WHO Disability Assessment Schedule WHODAS 2.0.* 2010, Geneva: World Health Organisation.

81. Knaus, W.A., et al., *Apache-Ii - a Severity of Disease Classification-System.* Critical Care Medicine, 1985. **13**(10): p. 818-829.

82. Ely, E.W., et al., *Evaluation of delirium in critically ill patients: Validation of the Confusion Assessment Method for the Intensive Care Unit (CAM-ICU).* Critical Care Medicine, 2001. **29**(7): p. 1370-1379.

83. Shapiro E, L.b., *Early EMDR intervention (EEI): A summary, a theoretical model, and the recent traumatic episode protocol (R-TEP). .* Journal of EMDR Practice and Research,, 2008. **2**: p. 79-96.

84. Shapiro, F., *Eye movement desensitization and reprocessing: Basic principles, protocols, and procedures, 2nd ed*. Eye movement desensitization and reprocessing: Basic principles, protocols, and procedures, 2nd ed. 2001, New York, NY, US: Guilford Press. xxiv, 472-xxiv, 472.

85. Laub, B., Weiner, N., *A developmental/integrative perspective of the recent traumatic episode protocol.* Journal of EMDR Practice and Research, 2011. **5**: p. 57-72.

86. Andrews, B., et al., *Delayed-onset Posttraumatic stress disorder: A systematic review of the evidence.* American Journal of Psychiatry, 2007. **164**(9): p. 1319-1326.

87. Shapiro, E. and B. Laub, *Early EMDR Intervention Following a Community Critical Incident: A Randomized Clinical Trial.* Journal of Emdr Practice and Research, 2015. **9**(1): p. 17-27.

# **Appendix One:**

## The Recent Traumatic Episode Protocol (R-TEP): An Integrative Protocol for Early EMDR Intervention (EEI)

Elan Shapiro and Brurit Laub

**Early EMDR Intervention (EEI)**

The question of how early to intervene with EMDR in the face of natural and man-made disasters has been an important part of the dialogue of those working in this field. As a result of the human beings suffering in the wake of these catastrophes, a number of ideas have ensued and new ways to work with the pain and anguish have been explored. Whereas the majority of people who experience a significant trauma will recover spontaneously, there is often prolonged suffering and about one-third may be left with enduring distressing clinical or subclinical symptoms of posttraumatic stress disorder (PTSD) and other psychiatric disorders (National Institute for Clinical Excellence [NICE], 2018).

Early EMDR intervention (EEI), before consolidation of the memory has taken place, may reduce associative connections to past traumas, preventing the accumulation of traumatic memories. It may also enhance adaptive associations, promoting adaptive integration reflected in self-affirmation, coping, resilience, and other measures of “post-traumatic growth.” Therefore, early EMDR intervention should be considered following a significant trauma. How and when to intervene with EEI most effectively and whether it can thereby reduce the incidence of PTSD and other disorders that can follow trauma are among the challenges that need to be studied empirically.

Informed by the work of Francine Shapiro, Roger Solomon, and all of the friends and colleagues in the field who have contributed to the evolution of their thinking and practice and following clinical and empirical experience with early EMDR intervention in the wake of the 2006 Lebanon war, the authors have observed that the existing EEI protocols appear to focus on certain aspects or parts of the traumatic episode along an approximate time line continuum following a trauma, in accordance with the *Diagnostic and Statistical Manual of Mental Disorders (DSM-5)* [23] *.*They concluded that the unfinished processing of recent traumatic events may require a broader approach than existing early EMDR intervention (EEI) protocols provided.

Looking at the existing protocols, Shapiro and Laub [83] suggest that the earliest interventions (e.g., emergency room protocols) that use elements of EMDR, such as Bilateral Stimulation (BLS), are primarily used for calming and stabilization for Acute Stress Response (ASR). The EMD Protocol is most effectively used for processing intrusive sensorimotor fragments. The protocol for Recent Traumatic Events (RTE) is used for processing an unconsolidated discrete event and the Standard EMDR Protocol is used to process memories that are already consolidated in a theme cluster. However, they suggest that the original traumatic incident and its aftermath may be conceived more like an ongoing *trauma continuum* while the experiences have not yet been consolidated. They propose a new protocol called the Recent-Traumatic Episode Protocol (R-TEP), which incorporates and extends the existing EEI protocols by providing a new comprehensive, integrative protocol. The R-TEP thus bridges the gaps left by previous protocols and facilitates a transition from the EMD and RE protocols to the Standard EMDR Protocol.

The R-TEP takes the wisdom of the Standard EMDR Protocol [84] and applies it in an adapted form for recent events to provide a comprehensive approach to Early EMDR Intervention. It is a protocol that adapts the EMD and the Recent Event Protocols within a newly conceived extended time perspective, termed here the “Traumatic Episode.” The Traumatic Episode (or T-Episode) comprises a number of targets of disturbing fragments and experiences (images, sensations, feelings, and thoughts) in the trauma continuum, from the original incident until the present, which need to be processed.

New theoretical conceptualizations of the process of memory consolidation, relating to Francine Shapiro’s Adaptive Information Processing (AIP) model [84] guided the development of the R-TEP. It is suggested that the stages of this process proceed hierarchically according to part/whole relations aiming toward adaptive integration (see Figure 12.1). This integrative sequence is of a broadening focus from the intrusive image/ sensation fragment to the event, to the episode that includes many events, to the theme, and to the identity that is comprised of clusters of themes. When a part (such as an intrusive fragment) is stuck (blocked/dissociated or locked/re-experienced), the AIP system is disrupted and cannot move toward the next whole, and thus fails to reach integration. In- formation is transmitted at increasing levels of complexity, from the sensorimotor (sensory and somatic) to the experiential (sensorimotor and emotional) and to the meaning (sensorimotor, emotional, and cognitive) levels, perhaps matching the evolution of the brain. It is assumed that the AIP system moves toward integration dialectically via associative connections between the various opposites of the traumatic memory networks and the adaptive ones (horizontal dialectical movement) going through part/whole integrative sequences (vertical dialectical movement) [85] .

The R-TEP employs an adapted eight–phase structure, with some modifications for application to early EMDR intervention. These modifications are based on the fragmented nature of the memory, on the need for containment and safety, and the wider T-Episode time frame. The T-Episode is conceived as a continuum from the original incident to the present and anticipated future concerns.


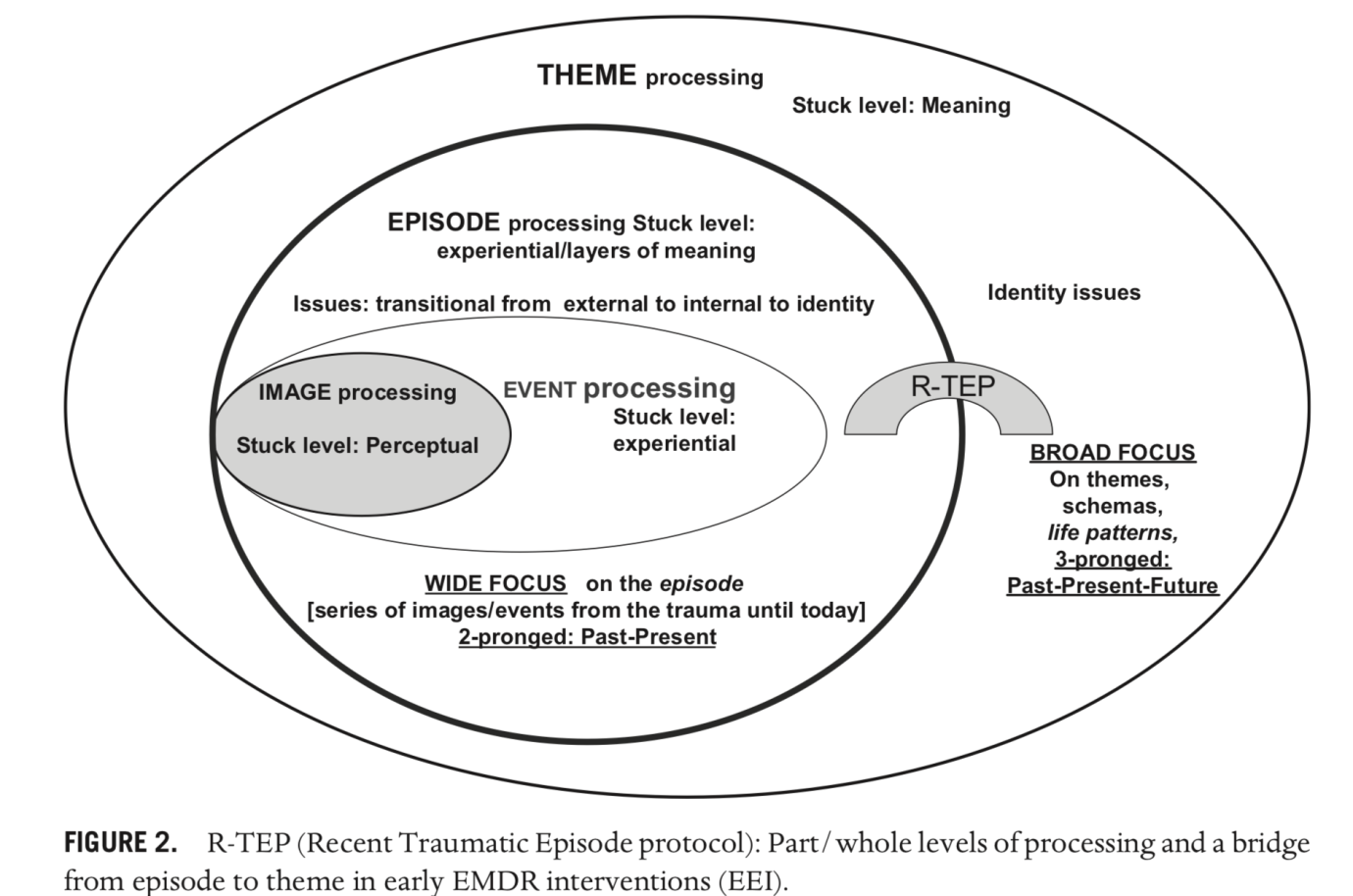


Main Issues in Early EMDR Intervention (EEI)

Clinical experience indicates that EMDR can be beneficial for alleviating excessive distress and complications in the weeks and months following critical events. However, there seems to be uncertainty and inconsistency among many clinicians about which protocols to use for Early EMDR Intervention and how and when to use them. Consequently, there is a need for a comprehensive model and set of guidelines in the EMDR practitioner’s toolbox to assist in approaching the prospect of EEI with more confidence and to generate research.

Issues to consider when working with EEI:

1. *Memory:* In recent trauma the nature of the memory is fragmented and not consolidated; it requires a different protocol.
2. *When to Intervene:* When there is distress, particularly when it is clinically significant, when to intervene is straightforward. However, when symptoms are sub- clinical, the question to ask is, “Is prevention to be considered?” Reference is made to the literature on delayed-onset and sensitization . [37, 86]
3. *Therapeutic Situation:* The nature of the situation for client and therapist is that there is an atmosphere of emergency or urgency that often results in high arousal or distress and sometimes avoidance; this requires a special attention to containment and safety.
4. *Therapy Contract:* The nature of the therapy contract may be unclear, and as a result professional and ethical standards may be compromised; this requires good practice guidelines. The R-TEP attempts to address these issues within the protocol as a comprehensive approach to EEI.

**The Recent-Traumatic Episode Protocol Features**

Main Features of R-TEP

1. A comprehensive approach to EEI: The eight phases.
2. An integrative approach to EEI:Incorporates adaptations of the EMD and RE protocols.
3. The Traumatic-Episode (T-Episode): This is a newly conceived trauma continuum time frame.
4. The Google-Search (G-Search): This is a procedure for scanning and identifying targets of disturbance or Point of Disturbance (PoD) within the T-Episode.
5. “Telescopic Processing”: Suggests three optional strategies for the processing in Phase 4 (Desensitization) for a contained intervention with varying boundaries for the chains of associations. Advocating a current trauma focus, the EMD strategy provides a narrow focus on the disturbing fragment; the EMDR strategy enables a broader focus on the current trauma episode; or (only if necessary and with client consent), the EMDR strategy that relates to the whole of life experiences.
6. Special attention to containment and safety.
7. Maintaining standards of good practice.
8. Theoretical underpinning.

**Appendix 2:**

**CovEMERALD Online EMDR R-TEP for survivors of Covid-19 relayed critical illness:** Template for intervention description and replication (TIDieR) checklist and guide [75].

1. **Brief name**: EMDR (Eye Movement Desensitisation and Reprocessing) R-TEP (Recent Traumatic Episode Protocol)
2. **Why**: The Covid-19 pandemic has resulted in a significant number of patients being admitted to Intensive Care for life-saving treatment. Research has revealed that a significant proportion of patients who survive their stay at Critical Care develop complications in their mental health, which can include symptoms of post-traumatic stress disorder (PTSD), depression, and anxiety, with long-term negative effects for patients and their families. In addition, patients who have survived a coronavirus related disease experience significant and persistent psychopathologies. There are currently very few NHS services that offer post-critical care support for patients, and those who do offer such support tend to focus more on the physical element of rehabilitation rather than the mental health recovery.

Some studies have reported significant improvement in psychological health for survivors of trauma following Eye Movement Desensitisation and Reprocessing (EMDR) therapy. EMDR is used to treat psychological trauma by targeting the way a traumatic event is stored and processed in the patient’s memory. Using bilateral stimulation, the aim is to help the patient reprocess the events, changing a disturbing memory into one that is no longer emotionally distressing and is perceived by the patient to have taken its appropriate place in the historical past.

Comparison studies have shown that EMDR can be better than other therapies (e.g. Cognitive Behaviour Therapy; Prolonged Exposure) at reducing level of psychological complications in inpatient and outpatient settings. Use of EMDR has been recommended by guidelines from the National Institute for Health and Care Excellence in relation to treating PTSD symptoms [44] and it is gradually receiving increasing endorsement as an evidence-based psychological treatment for trauma and often ensuing anxiety and depression.

The recent traumatic episode protocol (R-TEP) is a version of EMDR, developed to help with the processing of traumatic events before the psychological damage becomes entrenched. Using EMDR R-TEP, an individual’s psychological trauma is addressed in a matter of a few therapy sessions, thus targeting the trauma in its very early stages. There is emerging evidence that EMDR R-TEP may be applicable to trauma treatment in survivors of critical care. A pilot study carried out in France used EMDR R-TEP sessions in emergency room patients, which led to significant reduction in PTSD symptoms compared to a 15-minute reassurance and control groups.

Social distancing guidelines and the potentially long-term nature of the Covid-19 epidemic require the adoption and robust testing of technological solutions, in order to assure access to best possible psychological care.

With a clear need to address post-critical care psychological complications, and emerging evidence of EMDR R-TEP’s effectiveness in reducing trauma levels in related populations, there is a compelling case to understand whether an online EMDR R-TEP intervention may be effective in reducing psychological complications in survivors of Covid-19 related critical illness in the UK.

1. **What (materials)**: The online EMDR R-TEP intervention requires the use of a R-TEP worksheet. The clinician and participant would progress through the worksheet in a gradual manner, following the 8-phase approach of the R-TEP. The worksheet is a crucial part of the intervention as the participant would often have to refer to it throughout the treatment process.

In addition, the following hard copy outcome measures will be used in the study:

- PTSD Checklist-Civilian Version (PCL-C)
- Hospital Anxiety and Depression Scale (HADS)
- Montreal Cognitive Assessment (MOCA)
- Health Questionnaire (EQ-5D-5L)
- World health Organisation Disability Assessment Schedule (WHODAS 2.0)
- Patient Generated Subjective Global Assessment Nutritional status (PG-SGA)
- Brief Resilience Scale (BRS)
- Wrist worn activity monitoring
- Subjective Units of Distress (SUDs)

1. **What (procedure)**: Twenty-six eligible participants from a UK critical care unit will be recruited for the study. After gaining their consent, a baseline assessment will be carried out through the outcome measures mentioned in point 3 above. Participants will then be referred to the Intensive Psychological Therapies Service in Poole where the online EMDR R-TEP intervention will be arranged. The intervention itself will involve 2-8 one-hour sessions in addition to a follow-up assessment at 6 months post-hospital discharge. A post-hoc process evaluation will be completed via semi-structured interviews with study participants, a representative sample of trial decliners, and clinical staff in order to gain qualitative data regarding the perceived efficacy of the intervention.

EMDR R-TEP is a comprehensive current trauma-focussed protocol for early EMDR intervention. The EMDR R-TEP has an 8-phase approach and usually requires 2-4 sessions, which can optionally be conducted on successive days [87] (Shapiro & Laub, 2014). In its essence, the EMDR R-TEP is an adaptation of EMDR for early intervention, integrating existing wisdom while addressing some additional issues of the trauma. The EMDR R-TEP conceptualises the traumatic event as a fragmented experience which has not yet been consolidated so no single image represents the entire event. The EMDR R-TEP enables the processing of a number of targets, which are aspects or parts of the event in order to facilitate integration and consolidation [84] .

The 8 phases of EMDR R-TEP include:

1. Client history: Obtaining information about the client’s previous pathology, exploring their severity, motivation and strengths as well as administering the IES-R trauma screen.
2. Preparation: using stabilisation exercises (e.g. 4 elements, Safe/Calm Place) followed by bilateral stimulation and scanning whilst the client is describing a traumatic episode.
3. Assessment: The client describes a target, a negative cognition and a positive cognition followed by a measurement of their subjective units of distress (SUD)
4. Desensitisation: doing sets of bilateral stimulation to gradually reduce the client’s subjective units of distress
5. Installation: involves the installation of a positive cognition, with the validity of that cognition being evaluated at regular intervals in-between more periods of bilateral stimulation
6. Body scan: the client is asked to notice body sensations while bringing the target trauma to mind, with any residual body tension being reprocessed by the clinician
7. Closure: ensures a strong closure to target processing and a return to the stabilisation exercises
8. Re-evaluation: the client’s subjective units of distress and the validity of their positive cognition are re-evaluated followed by a re-administration of the IES-R trauma screen
9. **Who provided**: The intervention will be delivered by experienced clinicians who have been trained in EMDR R-TEP (2-day training workshop) by the treatment developer (Elan Shapiro) and have completed Part I and Part II of basic EMDR training. These will include a Consultant Clinical Psychologist and Psychological Therapists who have expertise working with clients presenting with complex trauma and enduring mental health difficulties such as PTSD and Personality Disorder.
10. **How**: R-TEP will be delivered online, via Skype or Zoom, on an individual basis for each participant. This will be over the course of 2-8 sessions. Following completion of R-TEP, participants will be contacted through post for their 4-month follow-up in order to complete the repeat outcome measures.
11. **Where**: Once eligible participants have consented to participate in the study, they will be referred to the Intensive Psychological Therapies Service team located at Branksome Clinic in Poole (Dorset). Online EMDR R-TEP sessions (including follow-up) will take place using the participant’s preferred platform of Skype or Zoom, in accordance with NHS Digital guidance. The environment is remote from the scene of the trauma (i.e. hospital) and we are hoping that this would cause less distress to participants while they are engaging in the intervention. In addition, the use of an online platform will enable access to. Abroad population of patients who may be physically unable to travel to a psychological service clinic. Branksome Clinic is a tertiary service for outpatients, who present with complex trauma and enduring mental health difficulties, and is part of the Dorset HealthCare University NHS Foundation Trust. The service consists of a multi-disciplinary team of therapists from a variety of core professional backgrounds such as Clinical Psychology, Nursing and Occupational Therapy. All staff are professionally trained, post qualification, in a minimum of one therapy that is delivered at the service.
12. **When and how much**: The R-TEP intervention for this study is planned to take between 2-8 weekly sessions per participant. Each session will last 60-90 minutes. At 4-months post-hospital discharge all patients will be contacted via telephone, by NHS research nurses, in order to arrange a repeat of the baseline assessments completed following consent. Patients will be able to complete these assessment questionnaires by post or over the telephone.
13. **Tailoring**: The number of therapy sessions can vary on an individual basis depending on the participant’s severity of the identified trauma and how able they are to address it during treatment. This will be discussed with the treating clinician and mutually agreed prior to establishing the therapeutic framework of the intervention. Another aspect which can be tailored is whether the 3-month follow-up session is completed face-to-face or through telephone depending on the participant’s needs.
14. **Modifications**: None expected
15. **How well (planned)**: All clinicians who will deliver EMDR R-TEP in this study have been trained in the delivery of the intervention and will adhere to a standardised protocol of treatment. The number of therapy sessions can vary depending on each participant and this is an aspect of the study which can be difficult to control or plan in advance. We will be collecting adherence data as our primary outcome. These will inform the design of a future randomised controlled trial.
16. **How well (actual)**: to be reported following trial completion.

**Appendix 3: Patient questionnaires**
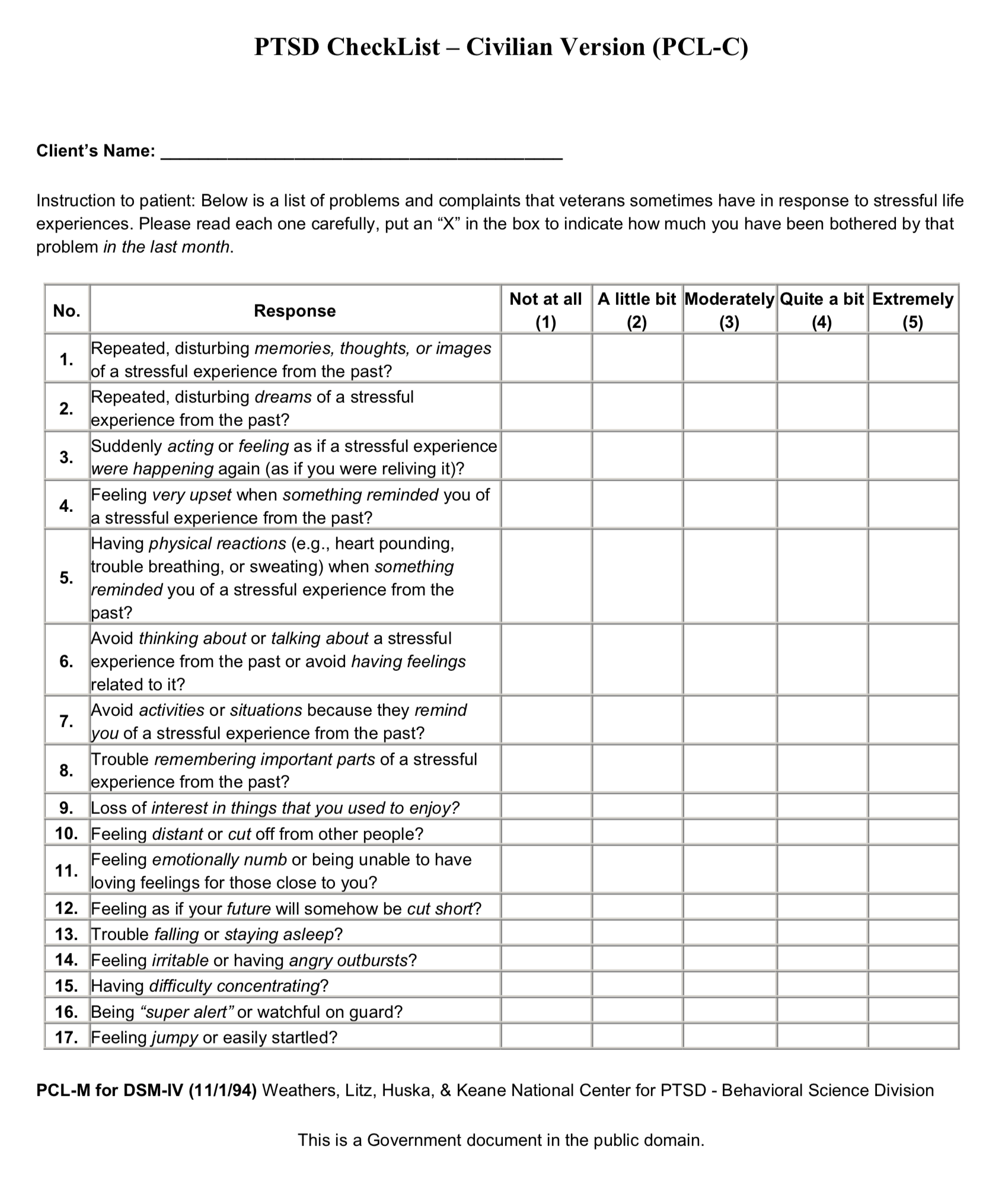


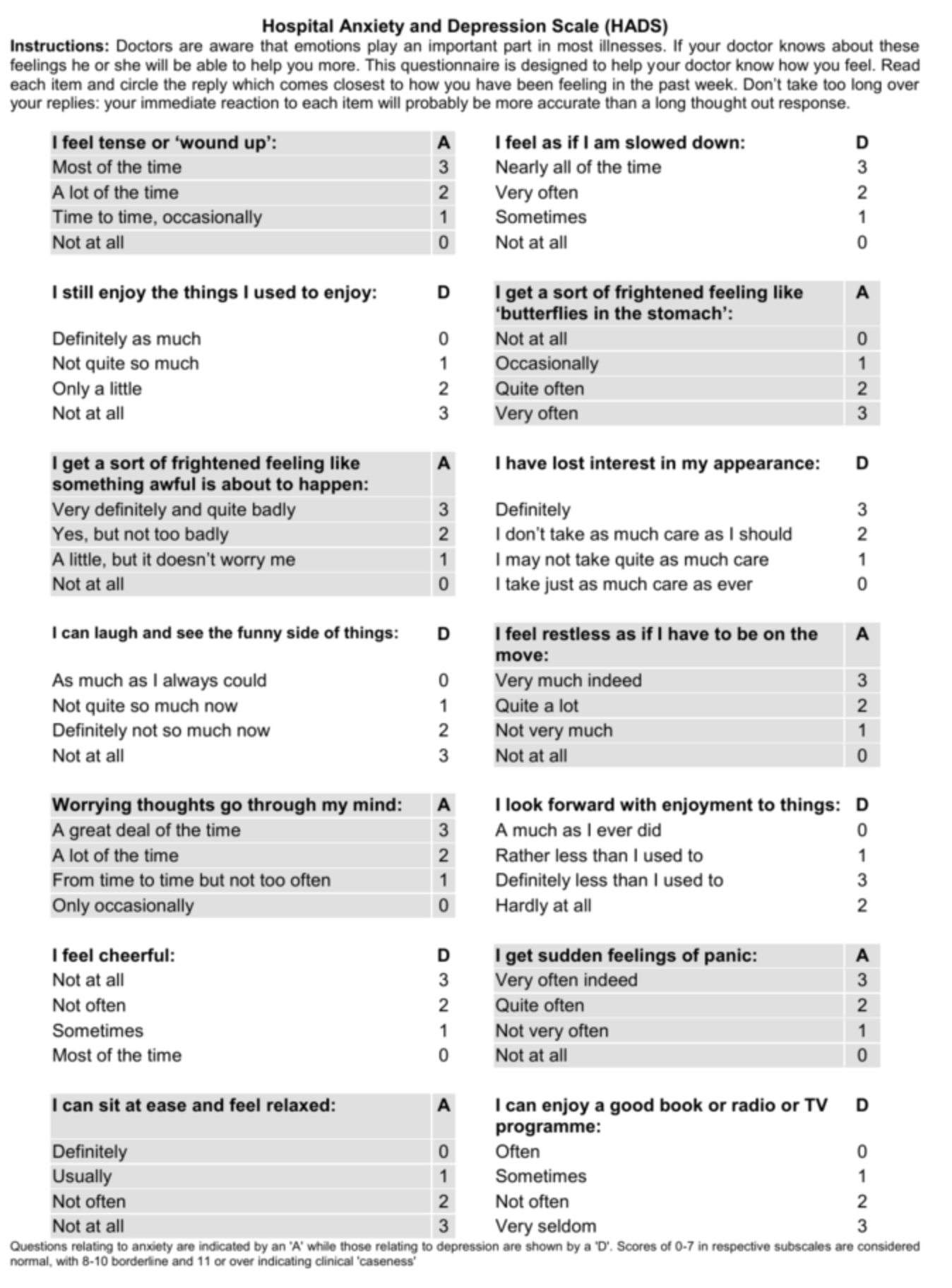


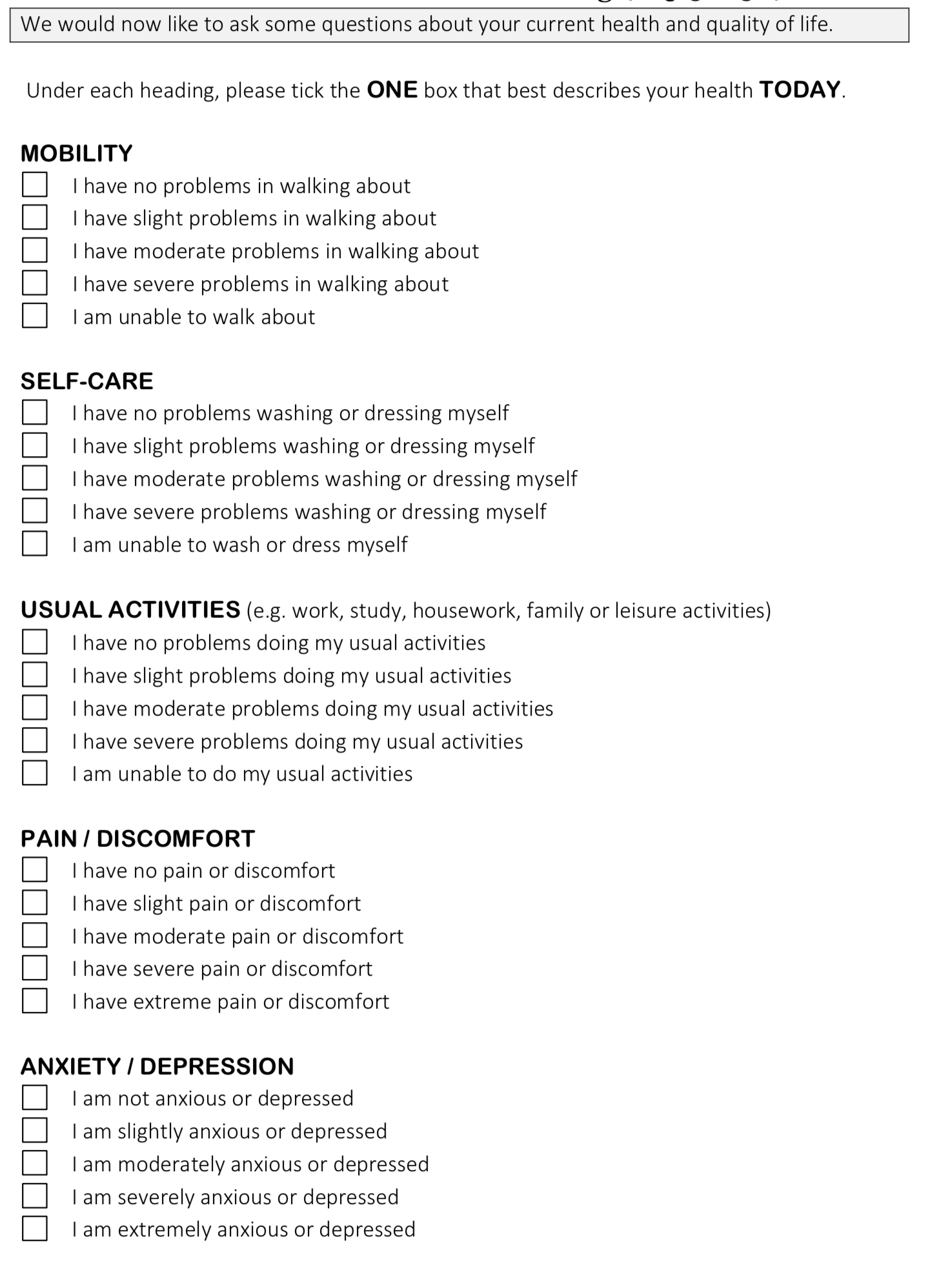

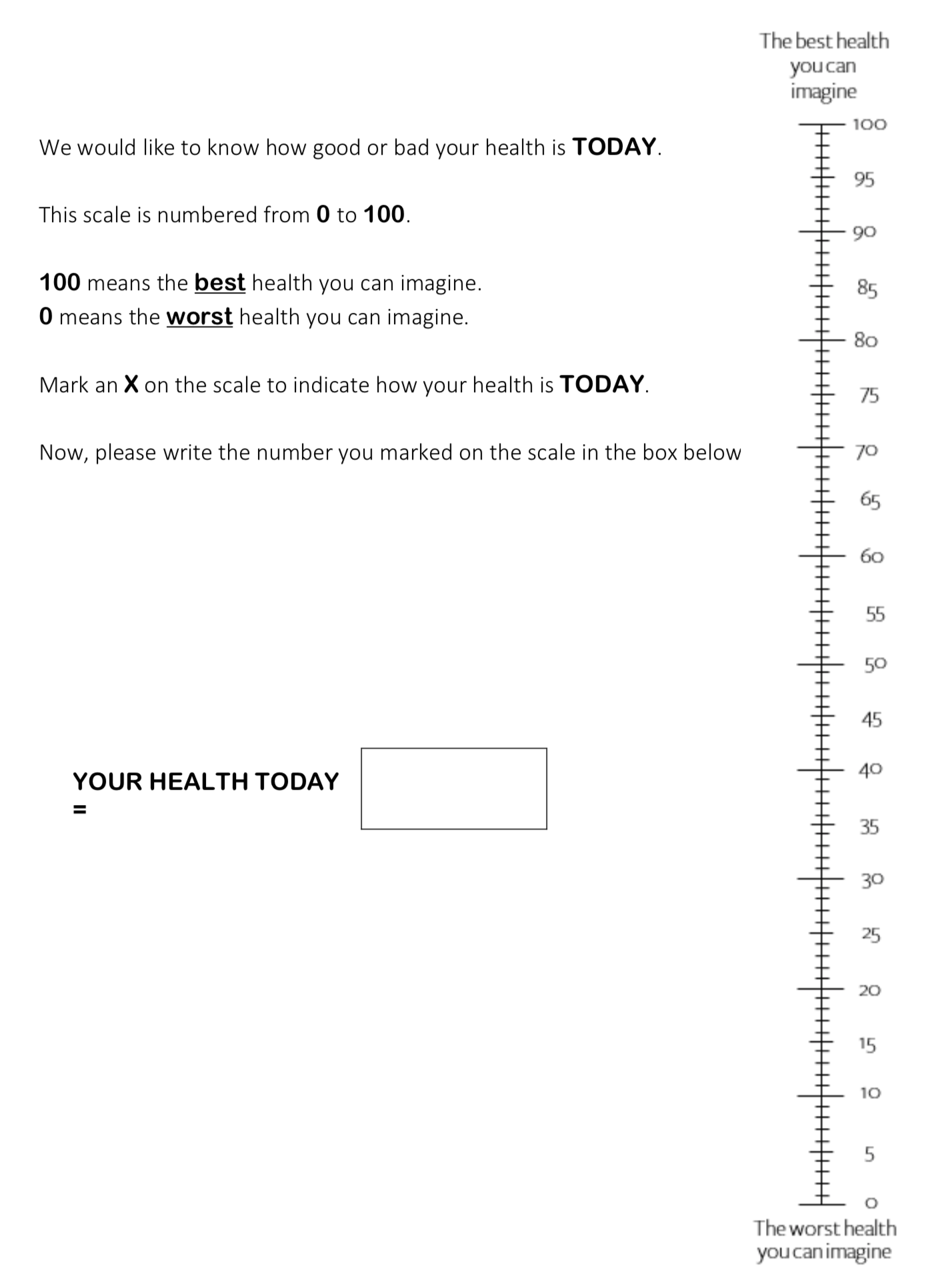

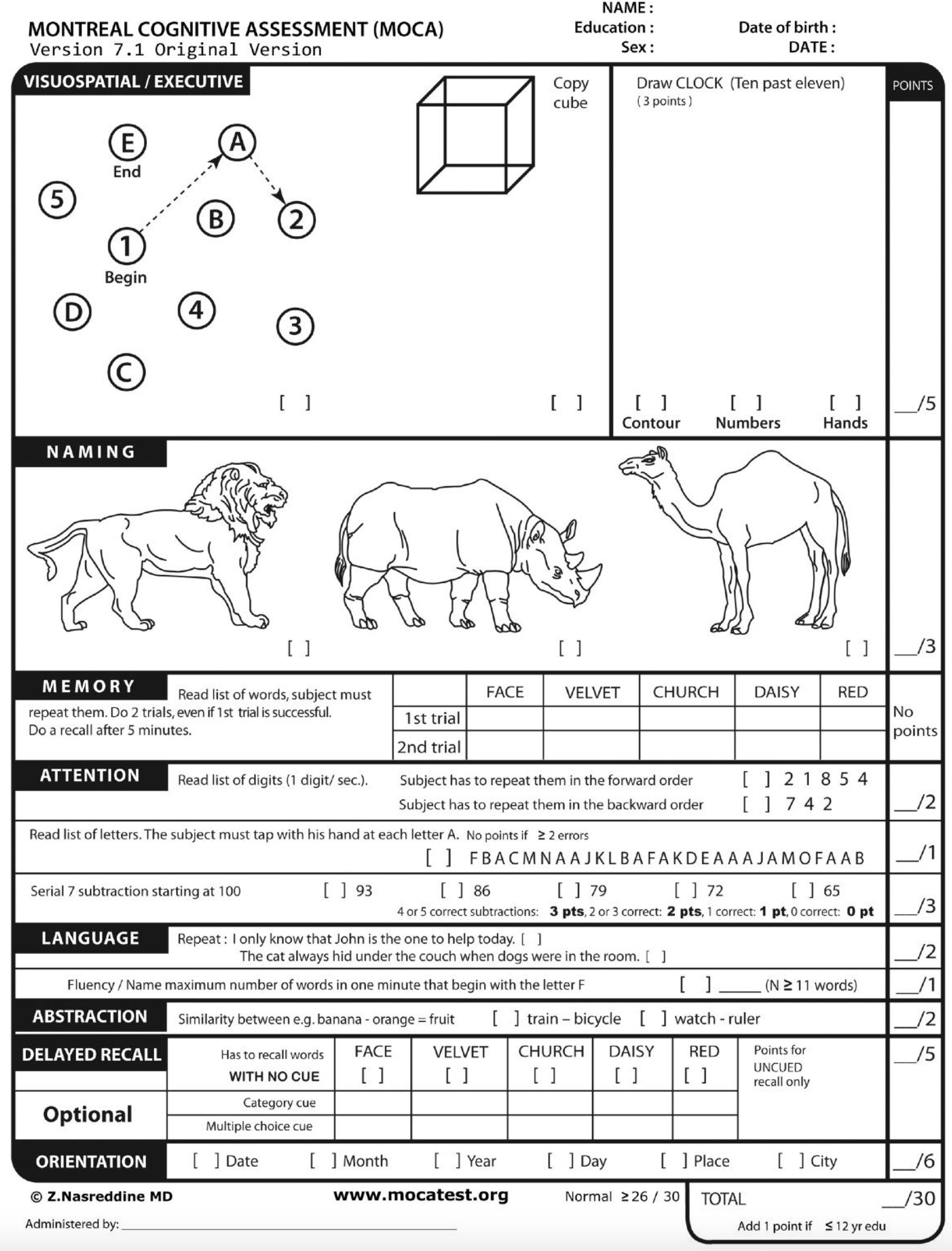


**
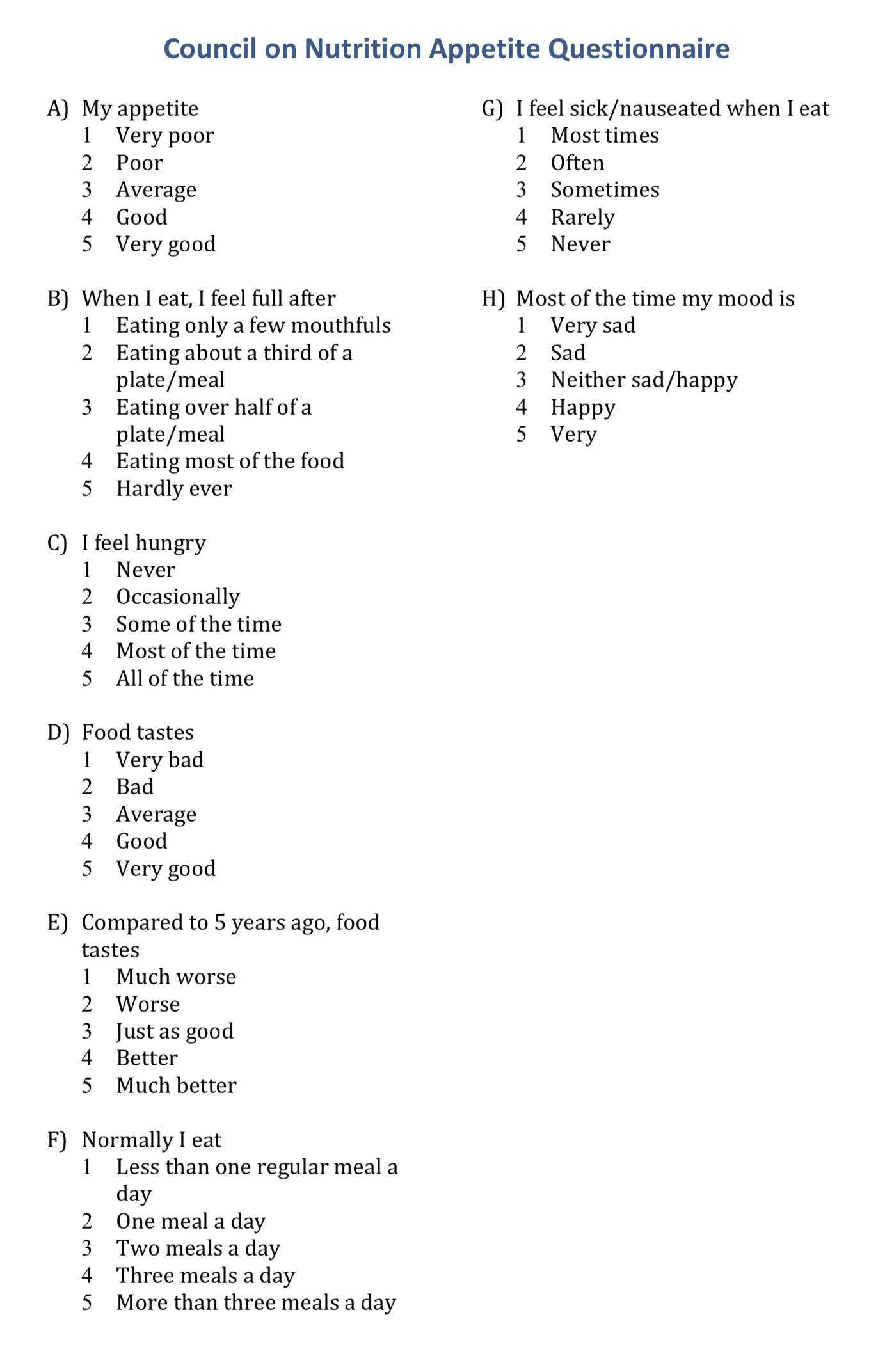
**

**
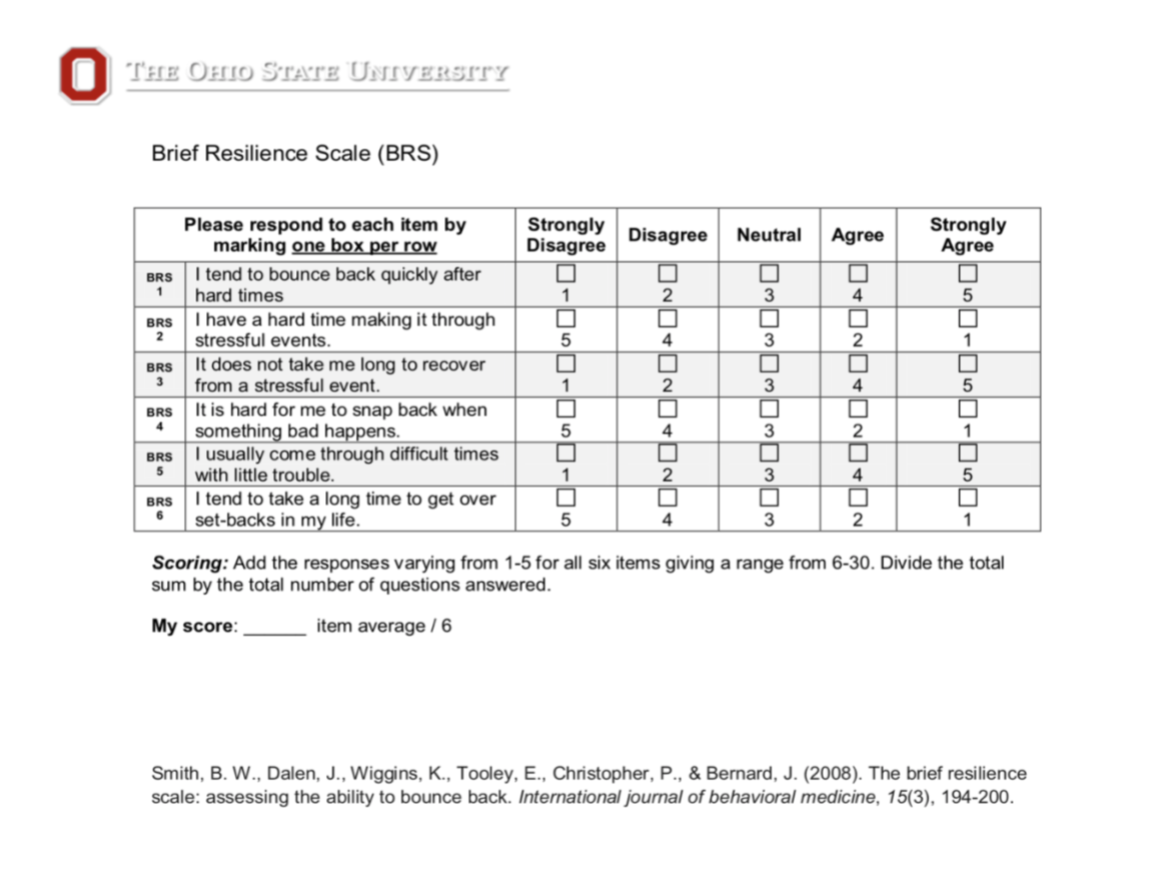
**
